# SnS Anodes with High Volumetric Capacity for Na‐ion Batteries and Their Characterization in Ether and Ester Electrolytes

**DOI:** 10.1002/smll.202503066

**Published:** 2025-08-26

**Authors:** Hui Wang, Yanan Sun, Thorsten Schultz, Katherine A. Mazzio, Vinita Ahuja, Yongchun Li, Volodymyr Baran, Norbert Koch, Philipp Adelhelm

**Affiliations:** ^1^ Department of Chemistry Humboldt University of Berlin Brook‐Taylor‐Str. 2 12489 Berlin Germany; ^2^ Joint research group CE‐GOBA Helmholtz‐Zentrum Berlin für Materialien und Energie (HZB) 12489 Berlin Germany; ^3^ Joint research group SE‐GHM Helmholtz‐Zentrum Berlin für Materialien und Energie (HZB) Hahn‐Meitner‐Platz 1 14109 Berlin Germany; ^4^ Deutsches Elektronen‐Synchrotron (DESY) Notkestraße 85 22607 Hamburg Germany; ^5^ Department of Physics Humboldt University of Berlin Zum Großen Windkanal 2 12489 Berlin Germany; ^6^ Center for the Science of Materials Berlin (CSMB) Humboldt University of Berlin Zum Großen Windkanal 2 12489 Berlin Germany

**Keywords:** anode, ether‐based electrolytes, SnS/t‐G, Sodium‐ion batteries, ultra‐flat discharge plateau

## Abstract

A current limitation to improving the volumetric energy density of Na‐ion batteries is the low density of the hard carbon(HC) anode. This problem can be solved by using high‐density, high‐capacity materials like SnS, which reacts with Na over a combined conversion and alloying reaction that theoretically provides 1022 mAh g^−1^ and 5335 mAh cc^−1^(materials level). Here, composites containing SnS and thermally activated graphite(t‐G) are prepared by ball‐milling and tested with different electrolyte solutions. Adding 5 wt.% of t‐G is sufficient to obtain significant improvements in capacity and cycle life, reaching 608 mAh g^−1^ initially and 439 mAh g^−1^ after 100 cycles. Even without calendaring, the obtained volumetric capacity of 283 mAh cc^−1^ (electrode level) is already on‐par with commercial HC electrodes. Moreover, ether‐based electrolytes are found to be superior to ester‐based electrolytes, enabling high storage capacity and cycle life. The reaction is investigated by  *operando*  X‐ray diffraction and *operando* dilatometry. The inferior performance in ester‐based electrolytes is found to be due to a larger polarization that largely prevents the alloying reaction that occurs close to 0 V. Over cycling, the conversion reaction becomes gradually inactive while the alloying reaction shows a much better degree of reversibility.

## Graphical abstract

High‐density SnS/t‐G composites enable sodium‐ion anodes with high gravimetric and volumetric capacities. A small amount of thermally activated graphite (5 wt.%) significantly enhances performance. The reaction is investigated using various methods, including *operando* XRD and *operando* dilatometry, ether‐based electrolytes facilitate reversible alloying near 0 V.

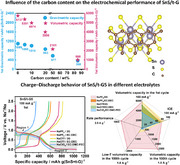

## 1 Introduction

Sodium‐ion batteries (SIBs) are currently being developed as a low‐cost alternative to lithium‐ion batteries (LIBs).^[^ ^1^, ^2^, ^3^ ^]^ Given the generally lower cell voltage of SIBs compared to LIBs, there is a great need to develop electrode materials with high storage capacity, suitable redox potentials, and long cycle life.^[^ ^4^ ^]^ Especially achieving high volumetric energy densities is an important task in the field of Na‐ion batteries, as the current first generation of commercial Na‐ion cells (≈250–300 Wh L^−1^) fall significantly below that of Li‐ion batteries (≈325 Wh L^−1^ for lithium iron phosphate (LFP) and values exceeding 580 Wh L^−1^ for nickel manganese cobalt (NMC chemistries). Currently, the energy density by weight and volume of commercial SIBs is typically 10–25% lower than that of commercial LFP batteries.

On the anode side, today's commercial SIBs rely on disordered carbon materials providing ≈300 mAh g^−1,^ which translates to a theoretical volumetric capacity of ≈450–600 mAh cc^−1^ (assuming a density of 1.5–2.0 g cc^−1^). Higher values can be obtained by using Na or metals such as Sn or Sb, yet all approaches also come with drawbacks such as dendrite formation, volume expansion, or increased costs. An alternative to alloying metals is metal chalcogenides.^[^ ^5^, ^6^ ^]^ Thanks to a combined conversion and alloying reaction, these materials exhibit very high theoretical capacities by weight and volume, while at the same time reducing the amount of metal (and hence cost) that is needed for reaching a certain capacity. The weaker metal‐sulfur bonds may also enable faster ion diffusion and less polarization compared to metal oxides.^[^ ^7^, ^8^ ^]^ SnS is a herzenbergite mineral with a GeS‐type layered structure, and its large interlayer distance (≈0.433 nm) may facilitate Na^+^ transport.^[^ ^9^, ^10^, ^11^ ^]^ Its stability against atmospheric water and oxygen eases storage and processing.^[^ ^12^, ^13^ ^]^ The theoretical reaction of SnS with Na can be separated into two main steps according to:^[^ ^14^, ^63^ ^]^

(1) $$\mathrm{Conversion}\;\;\mathrm{reaction}:\;\mathrm{SnS}+2{\mathrm{Na}}^{+}+2e^{-}⇌\mathrm{Sn}+{\mathrm{Na}}_{2}S$$

(2) $$\mathrm{Alloying}\;\mathrm{reaction}:\;\mathrm{Sn}+3.75{\mathrm{Na}}^{+}+\;3.75e^{-}\;⇌{\mathrm{Na}}_{3.75}\mathrm{Sn}$$

According to these reactions, SnS can react with a maximum of to form Na_3.75_Sn and Na_2_S. Reactions (1) and (2) provide a combined theoretical gravimetric capacity as high as 1022 mAh g(SnS)^−1^, i.e., more than three times compared to hard carbon. Assuming a bulk density of 5.22 g cc^−1^ of SnS, this corresponds to a theoretical volumetric capacity of 5335 mAh cc^−1^(SnS), which is ≈10 times more compared to hard carbon (details on the calculation can be found in the supporting information). The large capacity provided by conversion and alloying reactions comes with the challenge of volume expansion, which is even larger compared to the analogue reactions with lithium.^[^ ^15^ ^]^ While disordered carbons have a relatively low volumetric capacity, they have the advantage of showing only a minor expansion during sodiation. For example, dilatometry showed that hard‐carbon electrodes expand ≈2% during sodiation.^[^ ^16^ ^]^ In contrast, the expansion for SnS is theoretically 338% if the reaction goes to completion (details on the calculation can be found in the supporting information). Taking the sodiated state of the electrode, the theoretical volumetric capacity of (Na_3.75_Sn+Na_2_S) therefore amounts only to 1219 mAh cc^−1^, which is, however, still much larger compared to the 450–600 mAh cc^−1^ of today's hard carbons.

A strategy to mitigate volume expansion is to mix high capacity materials with carbon materials that buffer the volume change. For Sn/C composites (58 wt.% Sn and 42 wt.% C), the breathing could be reduced to 10–15%, while pure Sn theoretically expands by 420% during sodiation to form Na_3.75_Sn.^[^ ^17^ ^]^ While this approach is successful and also common in the field of developing Si/Graphite composites for LIBs,^[^ ^18^ ^]^ it is also immediately clear that a careful balance between the type and amount of carbon additive, the content of active material and the electrode porosity is needed to achieve a reasonable compromise between electrode capacity (by weight and volume), cycle life and cost.

Many examples show that the combination of SnS with carbon materials can greatly promote the electrochemical properties.^[^ ^19^, ^20^, ^21^ ^]^ Hou et al.^[^ ^22^ ^]^ prepared carbon‐coated SnS nanotubes through a templated method and used the composite as an anode for SIBs, where the reversible capacity and cycling stability were largely improved. Moon et al.^[^ ^23^ ^]^ attempted to use carbon layers to enhance the electrochemical performance of SnS nanoplates through a spin‐coating method, leading to a reversible capacity of 466 mAh g^−1^ after 50 cycles at a current density of 0.5 A g^−1^. Typically, the carbon constitutes a significant fraction of the electrode composites and thus reduces the capacity, but also often leads to a very porous electrode with low volumetric capacity. The addition of a large amount of carbon therefore, has a great impact on the volumetric capacity of the composite electrodes.^[^ ^24^ ^]^ At the same time, for large amounts of carbon, the contribution of carbon to the Na storage capacity can not be neglected anymore and should be included in the calculations.

The properties of anode materials are also very dependent on the electrolyte formulation. Early SIB research used ester‐based solvents as the electrolyte due to the experience gained from LIB research.^[^ ^25^, ^26^, ^27^ ^]^ An alternative to esters are ethers such as glymes.^[^ ^25^ ^]^ Diglyme (2G), for example, has a wide liquid temperature range (−64 to + 160 °C), good solvation properties, and a wide electrochemical window. Compared to esters, glymes are generally more stable at low potentials and less stable at high potentials. Diglyme typically leads to very flexible solid electrolyte interphases (SEIs) with low resistance and is therefore more compatible with many anode materials (Sb being an exception).^[^ ^28^ ^]^ In some cases, the reductive stability of diglyme even enables reversible co‐interalaction, such as in the case of graphite.^[^ ^29^, ^30^, ^31^, ^32^ ^]^ The wide liquid range and low viscosity also support a good low‐temperature ionic conductivity.^[^ ^33^, ^34^, ^35^, ^36^ ^]^ In a similar way, the conductive salt also plays an important role for achieving good performance. NaPF_6_ has become the most commonly used salt in SIBs, however, other salts such as NaOTf and NaClO_4_ are also being evaluated. Overall, while the use of SnS as the anode material appears attractive and has been studied in the past, there is a lack of understanding about the electrode reaction and the influence of the electrolyte. At the same time, in order to maximize the benefit of SnS, there is a need to prepare composites with low amounts of cheap carbon additives.

In this work, composites containing SnS and thermally activated graphite (SnS/t‐G) are prepared by a ball milling process. Adding only 5 wt.% of thermally activated graphite (sample denoted as SnS/t‐G5) is sufficient to significantly improve the electrochemical properties of the electrode. A comparison between ester (EC/DEC) and ether (2G) electrolyte solutions with NaPF_6_ as salt shows that the latter solvent enables a far better cycle life, reaching 439 mAh g^−1^ after 100 cycles (1.0 A g^−1^). Even at the low temperature of 0 °C, the SnS/t‐G5 electrode still delivers a high specific capacity of 340 mAh g^−1^ after more than 100 cycles (1.0 A g^−1^). Three‐electrode cell tests, *operando* XRD, and *operando* dilatometry results provide a comprehensive understanding of the electrode reaction and show that the electrolyte composition (especially the solvent) is the main reason why the ether‐based electrolytes show much better cycling stability. In ether‐based electrolytes, the high capacity observed over many cycles is due to the alloying reaction of Na with Sn, which contributes most of the capacity during long‐term cycling. More importantly, to explore the practical applications of SnS/t‐G anode, a full cell consisting of SnS/t‐G5 as the anode and Na_3_V_2_(PO_4_)_3_/C (NVP/C) as the cathode was assembled and showed stable cycling and excellent rate performance at both room and low temperatures.

## 2 Results and Discussion

### 2.1 Material Properties of SnS/Graphite Composites

**Figure 1:**
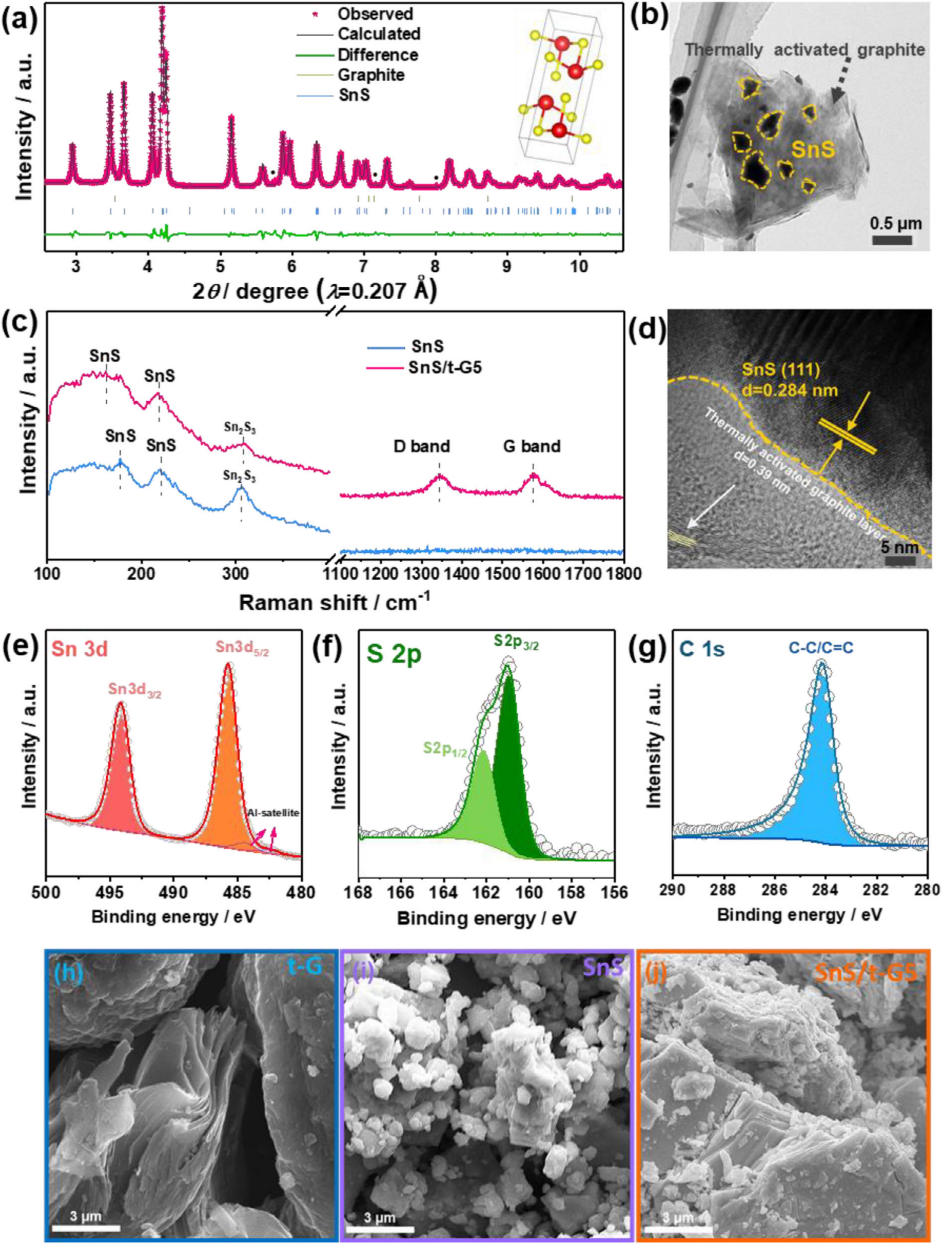
XRD data and Rietveld refinement of a) the SnS/t‐G5 composite. The solid circles indicate the Sn_2_S_3_ impurity. b) TEM and c) Raman spectra of pure SnS and SnS/t‐G5 materials. d) HR‐TEM images of SnS/t‐G5. The XPS spectra for e) Sn 3d, f) S 2p, and g) C 1s of SnS/t‐G5 material. SEM images of h) t‐G, i) SnS, and j) SnS/t‐G5.

Samples of SnS and SnS/graphite composites were prepared by ball milling from Sn, S, and graphite powders. In order to promote the interaction between graphite and the SnS powder, the graphite was pre‐activated by a thermal heat treatment at 630 °C for 10 h in air.^[^ ^37^ ^]^ The thermally activated graphite is denoted as t‐G. For the SnS/Graphite composites, the graphite content is given as a number, e.g. SnS/t‐G5 is a composite containing 95 wt.% of SnS and 5 wt.% of activated graphite. To obtain SnS through ball milling, the milling conditions were optimized as shown in Figures S1 and S2 (Supporting Information). It was found that the formation of SnS from the elements requires a high milling energy, as the compound does not form by hand grinding or low‐energy ball milling. To confirm formation of SnS and SnS/t‐G5 (here we show the characterization of the optimal ratio of 5wt.% t‐G‐SnS composites), high‐resolution synchrotron powder X‐ray diffraction (HRPXRD) was used to determine the constituent phases. Orthorhombic SnS with *Pnma* space group was indexed in both SnS/t‐G5 (**Figure** **1**a; Table S1, Supporting Information) and SnS (Figure S3 and Table S2, Supporting Information) by Rietveld refinement, where the SnS/t‐G5 was found to have lattice parameters of *a* = 11.201 Å, *b* = 3.985 Å, and *c* = 4.329 Å. Minor amounts of Sn_2_S_3_ impurities, indicated by weak characteristic peaks, were found in both samples. This indicates that a small amount of Sn is lost through the high‐energy ball milling process, e.g. by adhesion to the balls or the ball mill jar.^[^ ^38^ ^]^ Figure 1b shows the transmission electron microscopy (TEM) image of the as‐synthesized SnS/t‐G5 composite. SnS particles ranging in size from 200 to 500 nm are uniformly distributed throughout the t‐G matrix, which is further confirmed by elemental mapping analysis (Figure S4, Supporting Information). As shown in Figure 1d, the high‐resolution transmission electron microscope (HRTEM) image further confirms the presence of SnS and t‐G, where the measured interplanar spacing (0.284 nm) matches well with the SnS (111) crystallographic planes, while the interplanar spacing of ≈ 0.39 nm is consistent with the t‐G.^[^ ^37^ ^]^

The materials were further studied by Raman spectroscopy. As shown in Figure 1c, both SnS and SnS/t‐G5 exhibit the characteristic Raman bands at 161 and 217 cm^−1^, which can be assigned to the vibrational modes of orthorhombic SnS.^[^ ^39^ ^]^ The Raman bands of the Sn_2_S_3_ impurity could be observed in both samples as well. Additionally, the SnS/t‐G5 samples show broad *D* (1342 cm^−1^) and *G* (1580 cm^−1^) bands, indicative for some disorder in the graphite structure.^[^ ^40^ ^]^ The XPS spectra of Sn 3d, S 2p, and C 1s (Figure 1e–g) in SnS/t‐G5 confirm the presence of SnS within the composite. The Sn 3d core level consists of one single doublet at 485.8 and 494.0 eV, which is related to the Sn 3d_5/2_ and 3d_3/2_ in SnS.^[^ ^41^ ^]^ The same way the S 2p core level consists of only one doublet with binding energies at 161.3 and 162.6 eV, corresponding to the S 2p_3/2_ and S 2p_1/2_ of S^2–^ in SnS, respectively. The C 1s peaks for pristine t‐G and SnS/t‐G5 are virtually identical, demonstrating no chemical bond formation. Sn_2_S_3_ is found in the Sn 3d and S 2p spectra of pure SnS from the XPS spectra (see Figure S5, Supporting Information),^[^ ^41^ ^]^ which is consistent with the XRD and Raman results. No Sn_2_S_3_ was observed in the XPS spectra of SnS/t‐G5 samples. This could be attributed to Sn_2_S_3_ being integrated with t‐G during the composite process, and is not exposed on the surface of the material, limiting its detection by XPS. As shown in Figure 1h–j, the SEM images clearly show the morphological differences between SnS, t‐G, and the SnS/t‐G5 composite. In the SnS/t‐G5 composite, SnS particles are anchored to the surface of t‐G. Combined with the TEM results, this composite architecture forms a continuous layered network provided by t‐G, offering favorable pathways for electron transport and improved stability that can improve cycle life.

### 2.2 Influence of the Carbon Content on the Electrochemical Performance of SnS/t‐G

**Figure 2:**
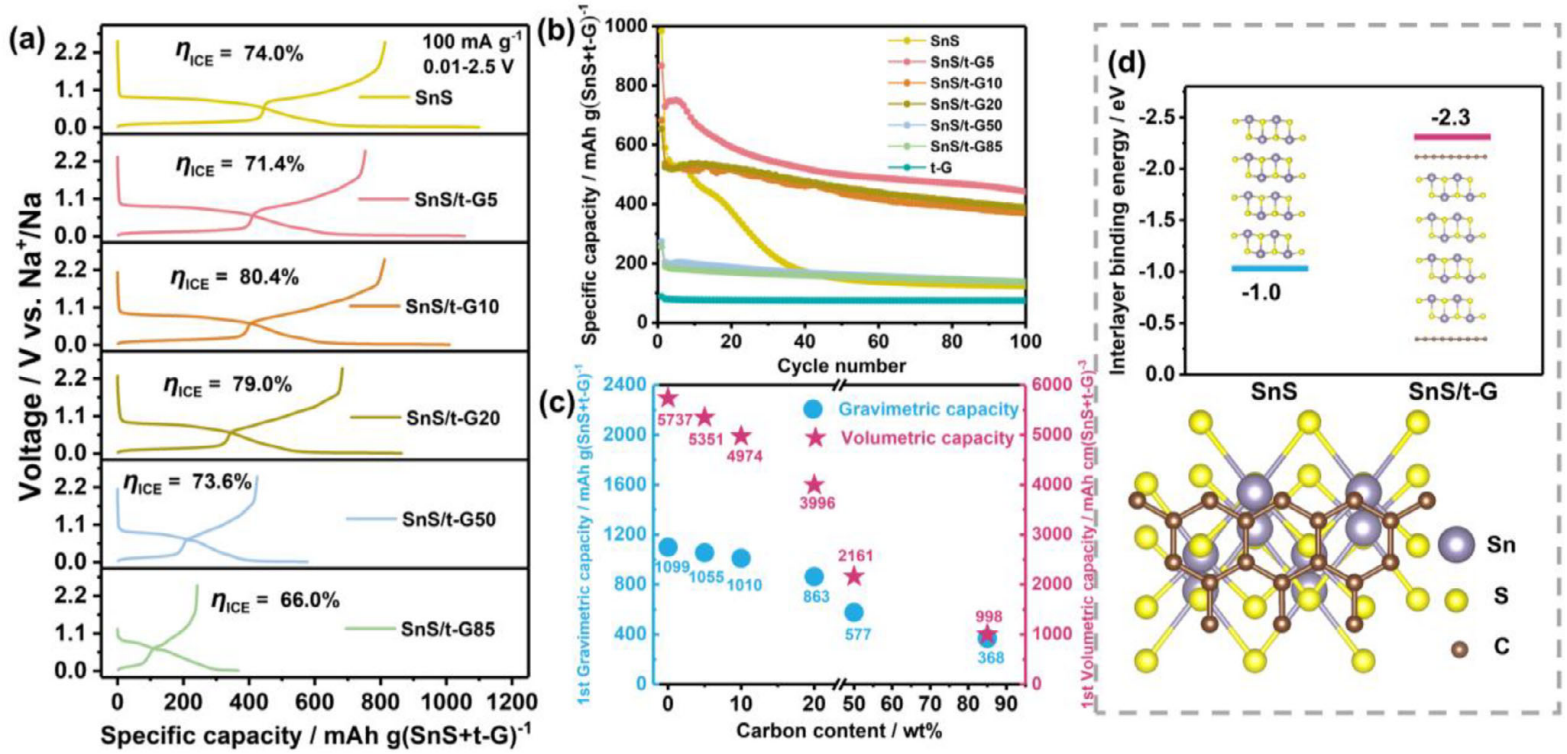
Electrochemical measurements and DFT calculations a) The initial galvanostatic charge/discharge curves of SnS/t‐G (0, 5, 10, 20, 50, and 85 wt.%) electrodes at a current of 100 mA g^−1^ in a voltage window of 0.01–2.5 V versus Na^+^/Na. b) Cycling performance of SnS/t‐G and t‐G electrodes at 1.0 A g^−1^ in a voltage window of 0.01–2.5 V versus Na^+^/Na. c) Gravimetric and volumetric capacity for SnS/t‐G electrodes, and values for the volumetric capacity refer to the amounts of SnS and t‐G, assuming their bulk densities. All measurements are done in two‐electrode cells with Na as the counter electrode and NaPF_6_/2G as the electrolyte. d) DFT calculations on the interlayer binding energy for SnS and for SnS/t‐G (approximated by four layers of SnS sandwiched between two graphenes).

To explore the effect of the amount of t‐G in the SnS/t‐G electrode on the battery performance, composites with different amounts of t‐G (0, 5, 10, 20, 50, and 85 wt.%), namely SnS, SnS/t‐G5, SnS/t‐G10, SnS/t‐G20, SnS/t‐G50, and SnS/t‐G85, respectively, were investigated. The voltage profiles of various composites in the first and second cycles are shown in **Figures** **2**a and S6 (Supporting Information). The initial discharge capacity decreases with increasing t‐G content, delivering 1099, 1055, 1010, 863, 577, and 368 mAh g^−1^ for SnS, SnS/t‐G5, SnS/t‐G10, SnS/t‐G20, SnS/t‐G50, and SnS/t‐G85, respectively. The measured initial discharge capacity of SnS is slightly higher than the theoretical capacity, which can be caused by side reactions and the resulting SEI formation. Figure 2b and Figure S7 (Supporting Information) show the long‐term cycling performance of various composites at a high current density of 1.0 A g^−1^. Although the capacity retention increases with increasing t‐G content, the capacity decreases accordingly, as expected. It is worth noting that the cycling stability of SnS can be largely improved by the addition of only 5wt.% t‐G, indicating that a small amount of t‐G is already sufficient to improve the cycling stability of SnS with minimal weight penalty compared to pure SnS. A further improvement in lifetime and (initial) Coulomb efficiency might be possible by optimization of the formation cycle.

Considering that the density of graphite (2.27 g cc^−1^) is only less than half the one of SnS (5.22 g cc^−1^), the volumetric capacity of the SnS/t‐G composite with only 5 wt.% t‐G (5351 mAh cc^−1^) is much larger than the other composites (see Figure 2c and Calculation b in Supporting Information). On the other hand, the volumetric capacity based on the whole electrode was also calculated and compared with the commercial hard carbon electrode.^[^ ^42^ ^]^ The SnS/t‐G5 material is unique in combining improved electrochemical properties and high volumetric capacity at a comparably low carbon content. In contrast, many SnS/carbon composites rely on excessive amounts of carbon, which reduces volumetric capacity and complicates electrode fabrication. The carbon coating provides an efficient electronic wiring while maintaining a low carbon content. We also calculate the volumetric capacity by accounting for electrode porosity (the calculation details are provided in Calculation 2, Supporting Information). The volumetric capacity of the SnS/t‐G5 electrode before calendering is ≈283 mAh cc^−1^, which is already comparable to commercial, calendared hard carbon electrodes (281 mAh cc^−1^). Note that the porosity of the SnS/t‐G5 after casting and drying is as large as 88% while the one of calendered hard carbon electrodes in the range of 28–45%. These values show that SnS/t‐G5 electrodes can lead to superior volumetric capacity values once the electrodes are further optimized through calendaring. Furthermore, the SnS/t‐G5 composite was synthesized by a facile ball milling process, avoiding the complex fabrication routes (e.g., multi‐step synthesis, template removal, etc.) used in other SnS/carbon systems, making it more feasible for practical applications. Although the capacity retention of SnS/t‐G5 needs improvement, e.g. by increasing the t‐G content, the much higher volumetric capacity compared to hard carbon remains a strong point (439 mAh g^−1^ for SnS/t‐G5 vs 330 mAh g^−1^ for hard carbon after 100 cycles).

In order to explore the reason why only a small amount of t‐G already stabilizes the cycling performance of SnS, theoretical calculations on the binding energies of layered SnS and SnS/t‐G were performed (Figure 2d and Table S3, Supporting Information). The interlayer binding energy of SnS (–1.0 eV) was calculated assuming bulk properties, i.e., assuming that the SnS extends infinitely in the *c* direction. Each interlayer binding energy reflects the real interlayer interaction strength in the bulk phase. For SnS/t‐G, a model was constructed based on the atomic ratio of the composite, in this case containing ≈5 wt.% carbon (as illustrated in the inset of Figure 2d). The interlayer binding energy in the model (–2.3 eV) reflects the binding strength between two layers of graphene and four layers of SnS. This value indicates that the interlayer interaction between SnS‐graphene is stronger compared to SnS‐SnS, meaning that intimate mixing between SnS and graphite is thermodynamically favored. Therefore, the SnS/t‐G5 electrodes can achieve the best balance between high capacity (both gravimetric and volumetric) and long cycle life. Moreover, studies have shown that the incorporation of SnS with carbon materials markedly enhances its density of states (DOS) near the Fermi level, which plays a crucial role in improving the capacity and cycle stability of the SnS/carbon‐based anode for sodium‐ion storage.^[^ ^43^, ^44^ ^]^ In subsequent investigations, we therefore utilized SnS/t‐G5 to explore the impact of various electrolytes in the electrochemical performance of the SnS/t‐G5 electrode.

### 2.3 Charge–Discharge Behavior of SnS/t‐G5 in Different Electrolytes

**Figure 3:**
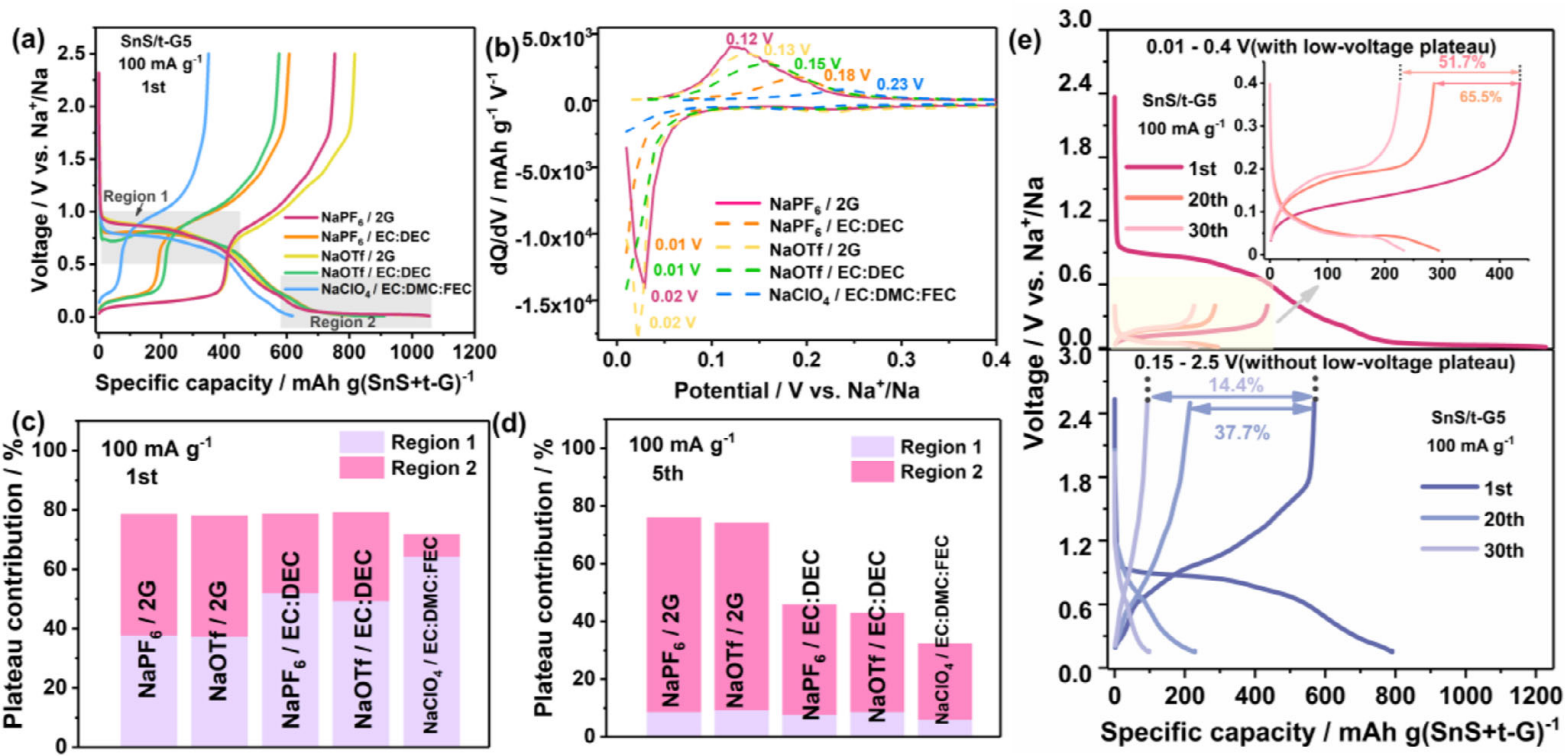
Na storage properties for different electrolyte formulations. The concentration was 1 M  in all cases a) The initial charge−discharge curves of SnS/t‐G5 electrode in different electrolytes in a voltage window of 0.01–2.5 V versus Na^+^/Na. b) Differential capacity versus voltage curves of the SnS/t‐G5 electrodes during the first cycle in different electrolytes in a voltage window of 0.01–0.4 V versus Na^+^/Na. c) Comparison of plateau capacity contributions of the first cycle and d) fifth cycles in different electrolytes in a voltage window of 0.01–2.5 V versus Na^+^/Na. The calculation formula for plateau capacity contribution is as follows: plateau capacity contribution = regional plateau capacity / total discharge capacity. e) The charge−discharge curves of SnS/t‐G5 electrode with NaPF_6_/2G electrolyte in different voltage windows after 1, 20, and 30 cycles. (All electrochemical measurements for this figure were performed at a current density of 100 mA g^−1^).

To understand the effect of electrolyte composition on the electrochemical properties, SnS/t‐G5 electrodes were evaluated using half cells with sodium as the counter electrode in different electrolytes (See Table S4, Supporting Information for details of the electrolytes). The capacity was calculated based on the mass of the SnS/t‐G composite. As shown in **Figure** **3**a, the SnS/t‐G5 electrodes showed similar electrochemical behaviors in the ether‐based electrolytes with different salts, where SnS/t‐G5 with NaPF_6_/2G and NaOTf/2G electrolytes delivered high discharge capacities of 1055 and 1052 mAh g^−1^ for the first cycle, with an initial Coulomb efficiency (ICE) of 71.4% and 77.7%, respectively. While for the ester‐based electrolytes, NaPF_6_/EC:DEC, NaOTf/EC:DEC, and NaClO_4_/EC:DMC:FEC delivered initial discharge capacities of 862, 911, and 618 mAh g^−1^, with an ICE of 70.5, 63.2, and 56.7% respectively. The higher ICE in both ether‐based electrolytes can be attributed to fewer side reactions and the formation of a more stable SEI layer compared to ester‐based solvents.^[^ ^45^ ^]^ All discharge curves show a plateau at ≈0.8 V versus Na^+^/Na, which corresponds to the conversion reaction of SnS (SnS + 2Na^+^ + 2e^−^ → Na_2_S + Sn), denoted as Region 1. It is worth noting that the voltage profile for the ether‐based electrolytes has a flat and well‐defined plateau at 0.03 V versus Na^+^/Na, which can be attributed to the alloying reaction 3.75Na^+^ + Sn + 3.75e^−^ → Na_3.75_Sn, denoted as Region 2.^[^ ^14^ ^]^

After long‐term cycling (shown in Figure S8, Supporting Information), the low‐voltage plateau still remains while the plateau related to the conversion reaction has disappeared. Therefore, the low voltage plateau plays an important role in maintaining the electrochemical properties of the SnS‐based electrodes. Differential capacity versus voltage (d*Q*/d*V*) plots (Figure 3b; Figure S9, Supporting Information) further confirm the two‐step electrochemical behavior in the first charge/discharge process. It should be noted that the SnS/t‐G5 electrode with the NaPF_6_/2G electrolyte delivers the narrowest voltage gap between the discharge and charge processes, indicating fast kinetics of the SnS/t‐G5 electrode in the NaPF_6_/2G electrolyte.

To better understand the effect of the low voltage plateau, the capacity contribution from the slope and plateau regions has been separated and plotted for the first and fifth cycles for each region (Figure 3c,d; Figures S10, and S11, Supporting Information). Region 1 is defined as the conversion voltage plateau capacity from the voltage steady state until the voltage shows an obvious decreasing trend. Region 2 is defined as the plateau capacity of the alloying process from the voltage starting to show a stable trend until the complete discharge. The contribution of the regions to the capacity is quite different for the different electrolytes. While in the first cycle, both regions contribute to similar amounts to the capacity when ethers are used, region 1 clearly dominates in ester‐based electrolytes. This indicates that the alloying reaction is less accessible in cells with ester‐based electrolytes. After 5 cycles, the capacity contribution in Region 2 becomes dominant for all electrolytes, indicating that the alloying reaction is more reversible over cycling compared to the conversion process.

To more clearly illustrate the relevance of the low voltage plateau for achieving longer cycle life, the electrodes were cycled in different voltage windows with variations for the lower cut‐off voltage. As shown in Figure 3e, the SnS/t‐G5 delivers a superior initial discharge capacity with a more pronounced voltage plateau in the 0.01–0.4 V versus Na^+^/Na range (i.e., with the low‐voltage plateau). After 30 cycles, SnS/t‐G5 provides a capacity retention of 52% for the lower cut‐off voltage of 0.01 V, while much faster fading (capacity retention: 14%) is found when cycling between 0.15–2.5 V (i.e., without the low‐voltage alloying plateau).

**Figure 4:**
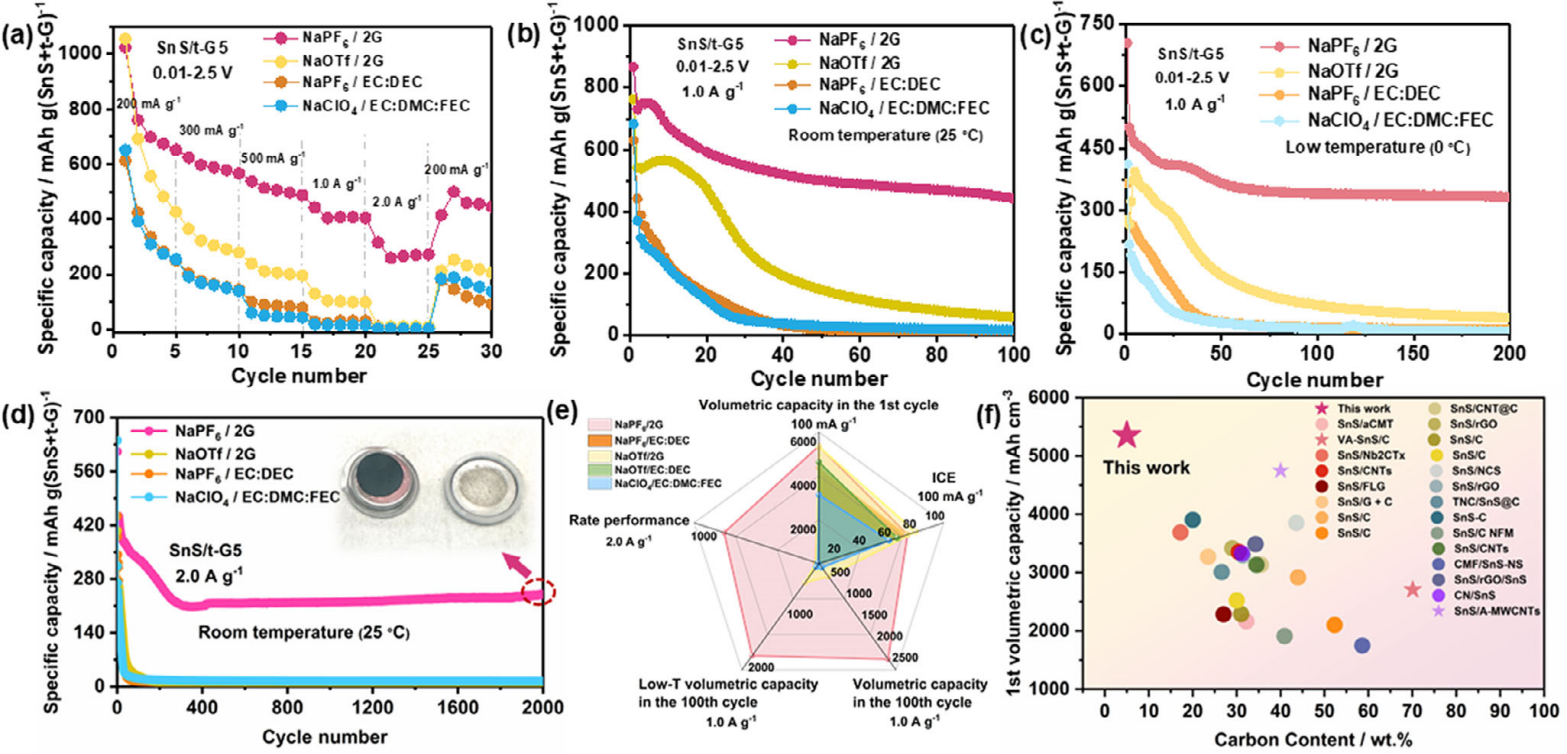
SnS/t‐G5 electrode in different electrolytes. a) Rate capability from 0.2 to 2.0 A g^−1^. b) Cycling performance at room temperature at 1.0 A g^−1^. c) Cycling performance at a low temperature 0 °C at 1.0 A g^−1^. d) The long‐term cycling performance at 2.0 A g^−1^_._ The inset shows the coin cell interior of a SnS/t‐G5 electrode after 2000 cycles. e) Schematic diagram of the comparison of different electrochemical properties in different electrolytes, including ICE at 100 mA g^−1^, initial volumetric capacity at 100 mA g^−1^, the volumetric capacity in 100th cycle at 1.0 A g^−1^, the volumetric capacity in 100th cycle at room‐temperature and low‐temperature at 1.0 A g^−1^, rate performance (2.0 A g^−1^). f) Comparison of the volumetric capacity (based on unsodiated SnS) of SnS/carbon electrodes in previously published studies and the present work. The use of ether‐based electrolytes and ester‐based electrolytes in the SnS/C electrode is indicated by solid stars and circles, respectively.

The rate performance and long‐term cycling stability of SnS/t‐G5 electrodes were further evaluated using different electrolytes and temperatures. Among the five electrolyte systems, the SnS/t‐G5 electrode in the NaPF_6_/2G electrolyte presents the best rate capability (**Figure** **4**a), with high capacities of 1025, 624, 538, 443, and 317 mAh g^−1^ at current densities of 0.2, 0.3, 0.5, 1.0, and 2.0 A g^−1^, respectively. In addition to the good rate capabilities, the SnS/t‐G5 electrode in NaPF_6_/2G electrolyte also exhibits outstanding cycling stability compared to the other electrolyte systems. At the high current density of 1.0 A g^−1^ at room temperature, the SnS/t‐G5 shows a much‐improved reversible capacity of 439 mAh g^−1^ with ≈72% capacity retention after 100 cycles (Figure 4b). Although the NaOTf/2G electrolyte delivers a discharge and charge capacity of 764/510 mAh g^−1^, the discharge/charge capacity quickly decays to less than 60/59 mAh g^−1^ after 100 cycles. In comparison, ester‐based electrolytes exhibit extremely poor cycling stability, with capacity decaying to ≈15 mAh g^−1^ after 100 cycles. The capacity at 200 mA g^−1^ after the rate capability test is lower than the initial capacity, which is mainly due to the intrinsic challenges of SnS‐based materials, such as large volume changes, intermediate phase transitions (e.g., Sn ↔ Na_3.75_Sn), and the general instability of the electrolyte at low voltages. The degradation mechanism can become more apparent at low currents and accumulate over cycling, hence causing differences in the capacity. This indicates that further optimization is needed. Nevertheless, it is already evident that the SnS/t‐G5 in NaPF_6_/2G exhibits favorable rate capability compared to the other electrolytes. Surprisingly, at low temperatures (0 °C), as shown in Figure 4c, the SnS/t‐G5 electrode in the NaPF_6_/2G electrolyte was also found to have superior electrochemical properties. A reversible capacity of 332 mAh g^−1^ was maintained after 200 cycles, i.e., only 8% capacity was lost from 50 cycles to 200 cycles, demonstrating the improved cycling stability of NaPF_6_/2G electrolyte even at low temperature. Remarkably, SnS/t‐G5 in the NaPF_6_/2G electrolyte shows good Na storage properties in terms of long cycle life. At a high current density of 2.0 A g^−1^, the SnS/t‐G5 electrode in NaPF_6_/2G electrolyte still maintained a reversible capacity of 240 mAh g^−1^ after 2000 cycles; the capacity dropped basically to zero much earlier for all other electrolytes (Figure 4d). After cycling, the SnS/t‐G5 electrode was disassembled from the cell, and it was found that the separator was still wetted, indicating that there are no severe side reactions observed during long‐term cycling. The slightly dark color observed on the surface of the separator is not caused by the dissolution of active materials. Instead, it is due to physical contact between the separator and the electrode during cell assembly. To better illustrate the difference in electrochemical performance of SnS/t‐G5 in different electrolytes, Figure 4e further summarizes and compares the key electrochemical properties, clearly showing the advantages of NaPF_6_/2G over cycle life. To further highlight the properties of the SnS/t‐G5 electrodes, the present results were compared to previously published SnS/carbon composites, see Figure 4f and Table S5 (Supporting Information). The SnS/t‐G5 composite exhibits an outstanding volumetric capacity even compared to other previously reported SnS/carbon composites. A comprehensive comparison on composition, cycling stability, and rate performance can be found in Figure S12 and Table S5 (Supporting Information). Considering the overall balance between electrochemical performance, cost of materials, and manufacturability, shows the potential benefits of SnS/t‐G5 as an anode material in SIBs. Overall, the systematic studies show that the properties of SnS as an active material highly depend on the electrolyte and electrode composition, with SnS/t‐G5 and NaPF_6_/2G being the most favorable combination.

### 2.4 Na Storage Mechanism in SnS/t‐G5 Electrode

**Figure 5:**
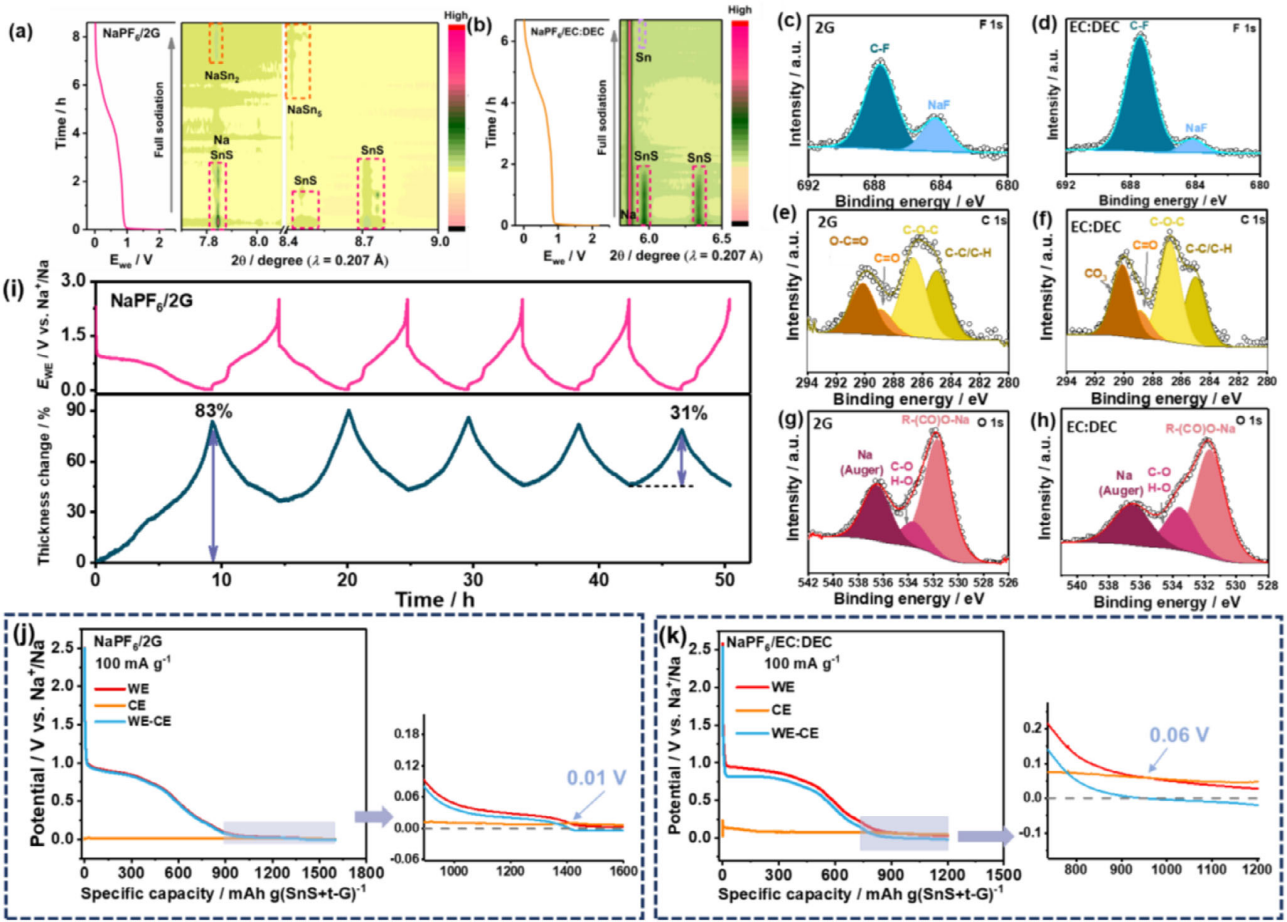
Contour plots of synchrotron‐based *operando* XRD measurements during the first discharge process of SnS/t‐G5 electrode using a) NaPF_6_/2G, full discharge capacity = 865 mAh g^−1^ and b) NaPF_6_ /EC:DEC electrolyte, full discharge capacity = 668 mAh g^−1^ The XPS spectra for c) and d) F 1s, e) and f) C 1s, g) and h) O 1s of NaPF_6_/2G and NaPF_6_/ EC/DEC during the first discharge process. (c) *Operando* ECD measurements with SnS/t‐G5 electrode as the working electrode in a three‐electrode cell with sodium as counter and reference electrodes, when using the NaPF_6_/2G, full discharge capacity = 926 mAh g^−1^. Initial discharge curves of SnS/t‐G5 electrode at 100 mA g^−1^ in the three‐electrode Swagelok‐type cell using (d) NaPF_6_/2G electrolyte, full discharge capacity = 1410 mAh g^−1,^ and (e) NaPF_6_/EC:DEC electrolyte, full discharge capacity = 940 mAh g^−1^.

To explore the underlying reasons for the more obvious low voltage plateau in ether‐based electrolytes, we selected NaPF_6_/2G and NaPF_6_/EC:DEC electrolytes for subsequent research and comparison. **Figure** **5** shows results for synchrotron‐based *operando* XRD, *operando* electrochemical dilatometry (ECD, 3 electrode cell), and Swagelok‐type three‐electrode measurements. The use of these methods requires cell designs that differ from the more simple coin cells and are more complex. For this reason, the discharge capacities are not always identical (see Figure text). However, the results reveal a much better understanding of the electrode reaction.

Figure 5a,b shows *operando* XRD contour plots for the initial sodiation of the SnS/t‐G5 electrode. For the NaPF_6_/2G electrolyte, the characteristic peaks of NaSn_2_ and NaSn_5_ from the alloying reaction can be observed, indicating that the alloying process is not complete, which is in line with expectations. Even though the intermediate phase does not completely transform into the end phase, this discrepancy can be attributed to the use of ether‐based electrolytes over ester‐based electrolytes, which causes a low‐voltage plateau. In addition, the side reaction, e.g. the formation of SEI in the first sodiation process, could be another cause of exceeding the theoretical capacity.^[^ ^46^, ^47^ ^]^ Formation of the end phase (Na_3.75_Sn) that would cause a large volume expansion, but was not observed by *operando* XRD.^[^ ^48^ ^]^ It is important to remember that amorphous phases may form, which can not be detected by XRD. Overall, however, partial sodiation reduces the overall volume expansion and hence may help to improve cycle life. For the EC:DEC‐based electrolyte, no alloying products could be observed, indicating that the alloying process is hindered in this electrolyte.

To gain an understanding of the components of the SEI, XPS was conducted for the NaPF_6_/2G and NaPF_6_/EC:DEC after first discharge. As shown in Figure 5c–h, the F 1s spectrum contains two peaks at ≈688 eV and ≈684 eV, belonging to P‐F and Na‐F, respectively. The C1s peaks at 284.8 eV (C─C/C─H), 286.6 eV (C─O), and 288.2 eV (C═O) mainly originated from some organic compounds derived from the reduction of solvents.^[^ ^49^ ^]^ The peak at 290 eV (O─C═O or CO_3_) originates from sodium alkoxides (RCH_2_ONa) for 2G, which is essential for the interfacial stability of the SnS/t‐G5 electrode. For NaPF_6_/EC:DEC, the peak at 290 eV in the C 1 s spectra is assigned to sodium alkyl carbonates (ROCO_2_Na).^[^ ^50^ ^]^ It can be observed from O 1 s spectrum and F 1 s spectrum that both samples contain NaF as inorganic components in SEI. The main different compositions of the SEI layers in glyme‐based electrolytes and carbonate‐based electrolytes are sodium alkoxides and polyethers versus sodium alkycarbonates and polyesters. These lead to the huge difference in the electrochemical performance of different electrolytes.

Although SnS undergoes a much severe volume expansion (theoretically ≈338%) at the material level, the volume change at the electrode level could be much smaller. Further insight into the reaction was obtained by *operando* ECD, where the change in thickness of the working electrode during cycling can be tracked. As shown in Figure 5c, the first sodiation process shows the largest electrode expansion (≈83%), while the electrode breathing tends to stabilize in the following cycles, with the thickness change of ≈31% in the fifth cycle. The stabilized thickness change of the electrode is advantageous for utilizing the high volumetric capacity of the SnS/t‐G5 material in practical applications.

In order to understand the different behavior of the electrode in the two different electrolyte systems, measurements in three‐electrode cells were conducted. This allowed to following the electrode potentials of both the working electrode (SnS/t‐G5) and the sodium counter electrode during cycling. All other experimental paramters remained the same as in the coin cell experiments. Figure 5d,e show the individual electrode potentials of the working (red line) and counter (orange) electrode as well as the cell voltage (blue line). The electrochemical behavior obtained in the three‐electrode cell is very similar to that of the two‐electrode coin cell, i.e., first a conversion reaction plateau appears, followed by a plateau due to the alloying reaction. When comparing the electrode potentials of the Na counter electrode for the different electrolytes, one can clearly see a larger polarization for the EC:DEC‐based electrolyte. This causes the cell voltage to reach the cut‐off potential before the alloying plateau is reached, i.e., the alloying mechanism in the EC:DEC electrolyte is largely not accessibly due to the polarization of the Na counter electrode. This is different from the 2G‐based electrolyte, where the polarization of the Na counter electrode is smaller and hence the alloying process takes place before reaching the 0.01 V cut‐off. Similar behaviors are observed in other ester‐ and ether‐based electrolytes (Figure S13, Supporting Information). Overall, this clearly shows that the increased polarization in ester‐based electrolytes is the main reason for why the alloying reaction can be only be observed in ether‐based electrolytes.

**Figure 6:**
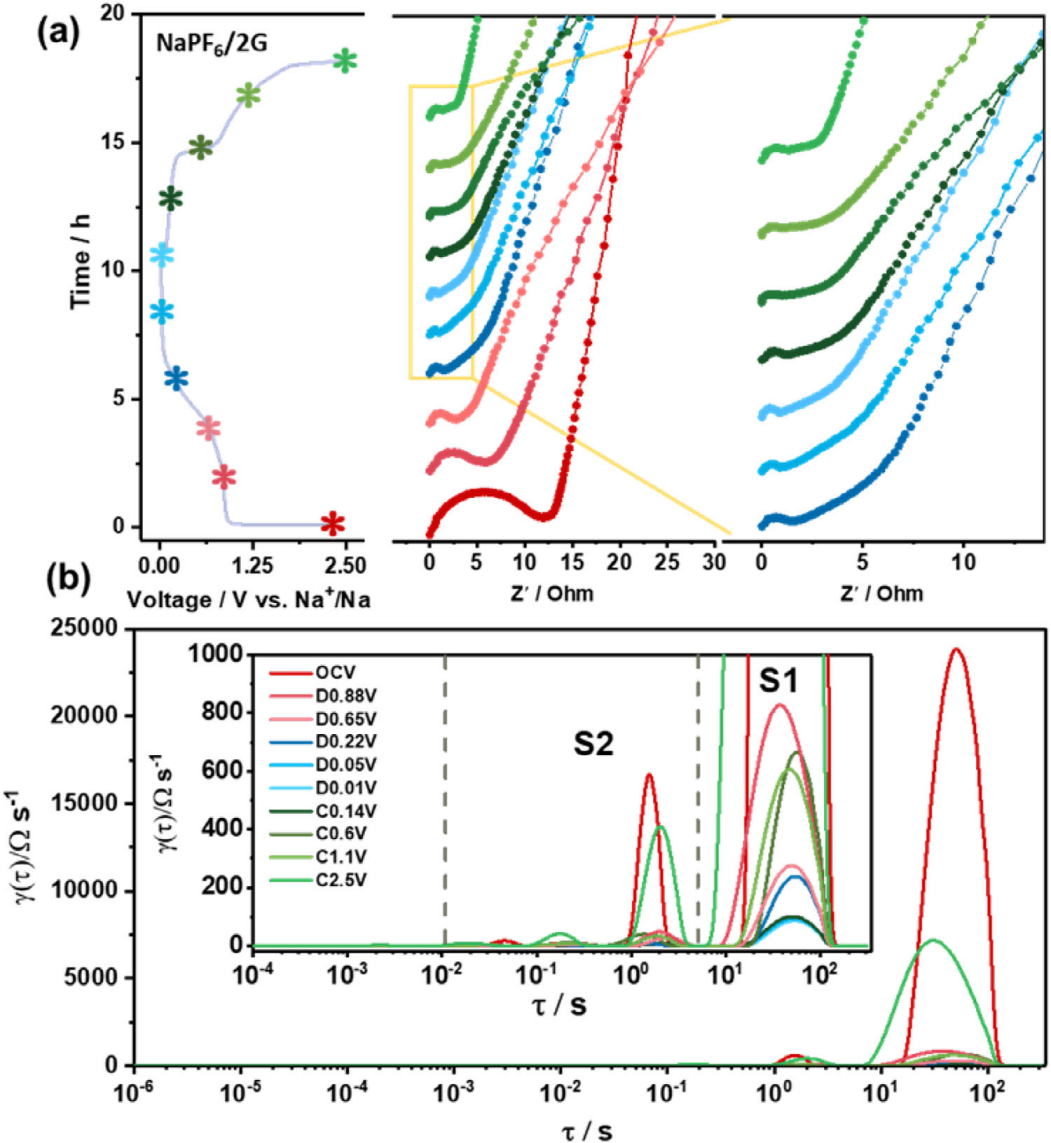
a)The in situ EIS spectra in NaPF_6_/2G. The initial cycle discharge and charge profile are plotted at the left side of each spectrum. b) DRT curves from in situ EIS measurements at different potentials.

In situ electrochemical impedance spectroscopy (EIS) was carried out to investigate the transport kinetic processes of the NaPF_6_/2G and NaPF_6_/EC:DEC at the electrode–electrolyte interface (**Figure** **6**; Figures S14–S16, Supporting Information). As shown in Figure 6a, the *R*_ct_ value in the 0.01 V (1.0 Ω) is much lower than that in the OCV state (12.2 Ω), which can be attributed to an activation process caused by gradual electrolyte penetration and metallic Sn formation. During the charging process, the *R*_ct_ still exhibits a relatively low resistance. As illustrated in Figure S14a (Supporting Information), the overall impedance of NaPF_6_/EC:DEC remains consistently higher than that of NaPF_6_/2G during sodiation and desodiation, indicating relatively slower charge transfer kinetics in the ester‐based electrolyte. As shown in Figure 6b, the distribution of relaxation times (DRT) analysis can be used to identify different kinetic processes with specific relaxation characteristics on time scales. The DRT plots are obtained from the in situ EIS data. The DRT curve can be divided into four distinct regions: the peak in Region S1 (Supporting Information) distributed above 10 s correspond to diffusion of Na^+^ in the electrode material bulk phase; the peak in Region S2 (Supporting Information) between 10^−2^ and 10 s represent charge transfer reactions; the peak in Region S3 (Supporting Information) signifies the impedance of SEI; the peak in Region S4 (Supporting Information) distributed between 10^−6^ and 10^−4^ s correspond to the contact resistance between the active material and the current collector.^[^ ^51^, ^52^ ^]^ During the sodiation process, the peak of Region S1 (Supporting Information) gradually decreases, reflects enhanced Na⁺ diffusion in SnS/t‐G5 electrodes, indicating enhanced electrolyte wettability at the electrode surface. The peak in Region S2 (Supporting Information) shows a significant decrease during the sodiation, indicating improved charge transfer kinetics. As illustrated in Figure S14b (Supporting Information), unlike NaPF_6_/2G, an obvious peak in Region S3 (Supporting Information) appears in the NaPF_6_/EC:DEC system, indicating the SEI layer formed in EC:DEC electrolyte is thicker or more uneven than that in the 2G electrolyte, resulting in a stronger impedance response.^[^ ^53^ ^]^ Although NaPF_6_/2G exhibits a higher initial impedance than NaOTf/2G (Figure S15, Supporting Information), DRT analysis reveals that it possesses a significantly lower resistance in the low‐frequency region (>1 s), suggesting more efficient interfacial ion transport and SEI formation, which likely contributes to its better cycling and rate performance. Note that while NaOTf/2G can work well in other electrode reactions,^[^ ^54^ ^]^ the poor performance in the case of SnS is likely related to the specific combination of salt and active material. These observations highlight the critical role of electrolyte composition in the impact on sodium storage kinetics. At this point, we also note that voltage sweep analysis is often used to distinguish between capacitive and faradaic contributions to the charge storage. This method, however, has clear limitations for battery electrodes, which is why it is not used.^[^ ^55^, ^56^ ^]^

### 2.5 Full Cells with Na_3_V_2_(PO_4_)_3_/C (NVP/C) as Cathode

**Figure 7:**
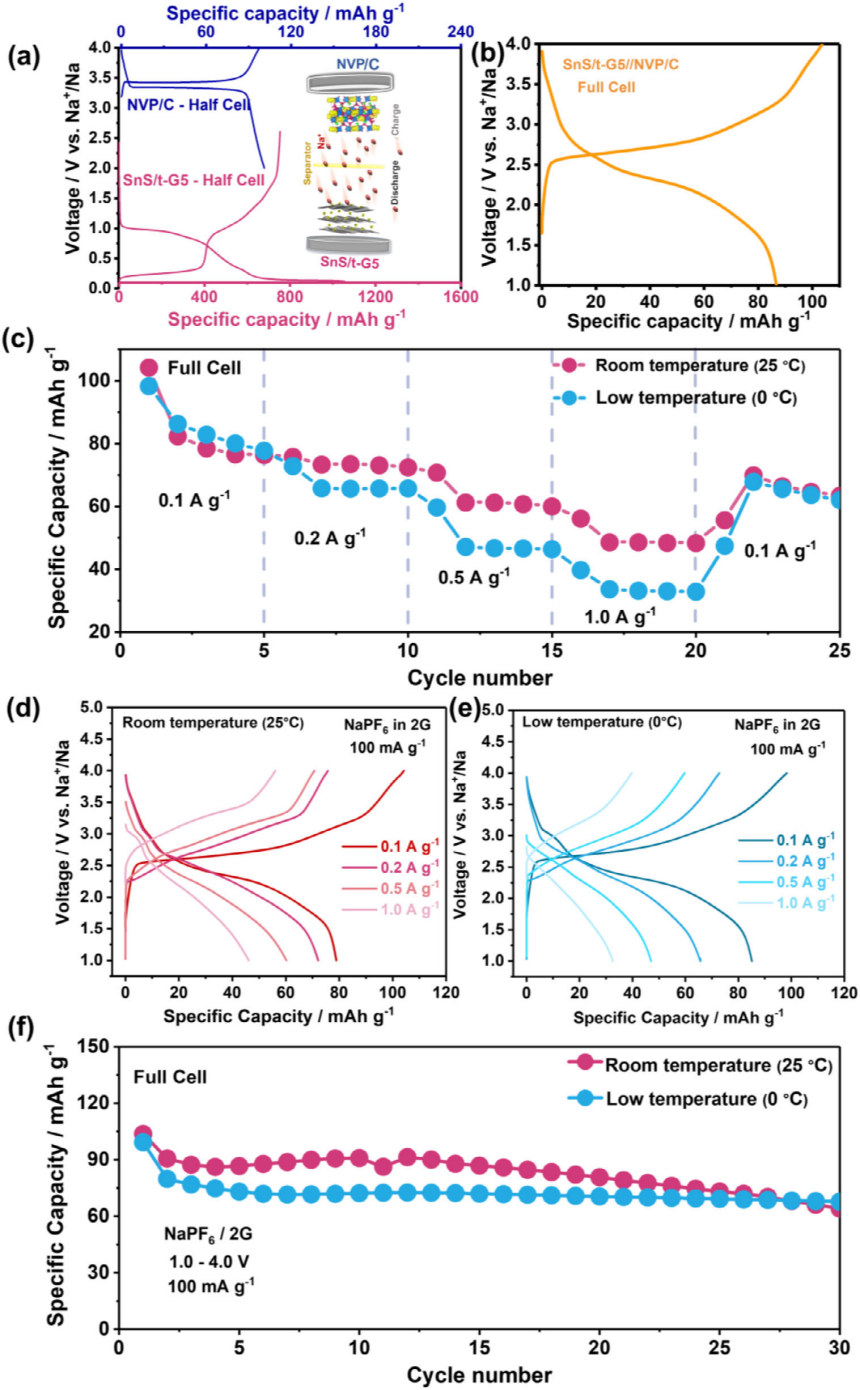
a) The initial charge−discharge curves of NVP/C cathode half cell at a current rate of 0.1 A g^−1^ in a voltage window of 2.0–4.0 V versus Na^+^/Na, and the SnS/t‐G5 anode half cell at a current rate of 0.1 A g^−1^ in a voltage window of 0.01–2.5 V versus Na^+^/Na. And a schematic of symmetric full cells using SnS/t‐G5//NVP/C. b) The initial charge−discharge curves of SnS/t‐G5 // NVP/C full cell at a current rate of 0.1 A g^−1^ in a voltage window of 1.0–4.0 V versus Na^+^/Na. c) Rate capability from 0.1 to 1.0 A g^−1^ at both room temperature and low temperature. The voltage profiles at different densities d) at room and e) at low temperatures. f) Cycling stability at 0.1 A g^−1^ both at room temperature and low temperature.

To explore the practical use of SnS/t‐G5 as anodes for Na‐ion batteries, full cells with Na_3_V_2_(PO_4_)_3_/C as cathode were assembled (denoted as SnS/t‐G5//NVP/C), see **Figure** **7**a,b. The structural characterization of NVP is displayed in Figure S17 (Supporting Information). The capacity ratio between the cathode and anode is designed as N/P = 1.5, taking into account the reversible capacities of SnS/t‐G5 anode and NVP/C cathode measured in half cell tests at a current density of 100 mA g^−1^, as well as the irreversible capacity losses during the first cycle. Figure 6c shows the rate performance and corresponding voltage profiles of the full cell from 0.1 to 1.0 A g^−1^. The initial charge and discharge capacities are 104 and 79 mAh g^−1^ (calculated based on the mass of the cathode), respectively, with an ICE of 76%. Moreover, the cell also exhibited good rate capability and cycling stability at 0 °C. The SnS/t‐G5//NVP/C cell showed good rate performance (Figure 7d), as demonstrated for a variety of current densities, providing reversible capacities of 79, 72, 60, and 46 mAh g^−1^, at 0.1, 0.2, 0.5, and 1.0 A g^−1^, respectively. Low temperature measurements at 0 °C were conducted as shown in Figure 7e. The SnS/t‐G5//NVP/C full cell delivered a specific capacity of 85 mAh g^−1^ at 0.1 A g^−1^ with a high ICE of 86.6%. This indicates that high capacity SnS electrodes may also be applicable at very low temperatures. The capacity could be restored to 65 mAh g^−1^ when the current density was returned to 0.1 A g^−1^. The cycling performance of SnS/t‐G5//NVP/C (Figure 7f) shows that the reversible capacity remains at 60 mAh g^−1^ after 30 cycles at 0.1 A g^−1^, corresponding to a capacity retention of 67%. For the same setup using NaPF_6_/EC:DEC as the electrolyte, the SnS/t‐G5//NVP/C demonstrated a larger initial irreversible capacity loss and a worse capacity retention of only 29% (Figure S18, Supporting Information). To provide additional insight into the long‐term performance, Figure S19 (Supporting Information) includes extended cycling data for half cells and full cells at both room and low temperatures. After 100 cycles, the SnS/t‐G5 electrode shows good cycling stability at low temperature (0 °C) in both half cells and full cells, further supporting the feasibility of SnS/t‐G5 electrodes in practical applications (Figure S19b, Supporting Information).

## 3 Conclusion

In summary, the use of SnS as an active material in Na‐ion batteries with thermally activated graphite as the electrode additive was explored. The most favorable composition with a high volumetric capacity of 5351 mAh cc^−1^ contained as little as 5 wt.% thermally activated graphite, while NaPF_6_/2G was identified as the most favorable electrolyte for this system. The electrode showed a markedly different behavior in ether‐ and ester‐based electrolytes. The reaction was studied by *operando* methods (XRD and ECD). In combination with the electrochemical results, it is found that the sodiation of SnS proceeds as expected over two major steps, a conversion reaction followed by an alloying reaction. The latter occurs at very low voltage and is very difficult to access when ester‐based electrolytes are used. Three‐electrode measurements showed that this is due to an increased polarization of the working (SnS) and counter electrode (Na) in ester‐based electrolytes.

Capacity and cycle life are better in ether electrolytes, where the alloying of Sn leads to a long, low‐voltage plateau. During cycling, this plateau was found to become the dominant storage mechanism, i.e., the conversion reaction become less reversible relative to the alloying reaction after many cycles.

A full cell with NVP/C as the cathode was assembled and could be cycled with only moderate capacity loss over 30 cycles at room temperature and at 0 °C, which additionally supports the favorable properties of the electrode‐electrolyte combination. Although the obtained cycle life is still inferior compared to that of hard carbons, the achievable capacity and electrode density are notably higher. The use of SnS with high density might therefore be a possible route to overcome the current low‐density bottleneck of hard carbon anodes for Na‐ion batteries.

## 4 Experimental Section

### Preparation of SnS and SnS/t‐G Materials

SnS was produced using the high‐energy ball milling technique (PBM, Fritsch Pulverisette 7). Tin powder (>99.8%, thermo scientific), sulfur powder (>99.5%, Alfa Aesar), and graphite (MTI Corp) were used as raw materials. Tin and sulfur powder were directly used without further purification, and weighed in an argon‐filled glove box from MBraun (H_2_O < 0.1 ppm, O_2_< 0.1 ppm) with a 1:1 molar ratio and stirred with a spatula to ensure evenly mixing. It was then placed into a 50 mL stainless steel jar in an argon filled glovebox. The samples were milled for 1 h in total with 10 min rest for every 4 min of milling at 500 rpm. The powder and ball weight ratio was 1:15. The obtained SnS powder was mixed with activated graphite at a weight ratio of 95:5 by ball milling for 1 h at 400 rpm in Ar atmosphere. The thermally activated graphite materials were synthesized according to the previous work.^[^ ^37^ ^]^ Considering the energy consumption of the ball mills in practical applications,^[^ ^57^ ^]^ 500 rpm as the appropriate speed was choosen.

### Preparation of the Electrode and Electrolyte

The electrodes for SnS/t‐G were be prepared from a mixture of 70 wt.% active material, 20 wt.% Super C65 (MTI Corp.) as conductive carbon additive and 10 wt.% Polyvinylidene fluoride (PVDF, >99.9%, MTI Corp) using N‐methyl‐2‐pyrrolidinone (NMP, >99.9%, Sigma–Aldrich) solvent as binder. The uniform slurry was then cast on an aluminum foil current collectors and then punched into 12 mm diameter electrodes, and pre‐driying on a hot plate. Subsequently, electrodes were dried under vacuum at 60 °C for 12 h using a Büchi oven. Electrolytes were prepared in an argon filled glovebox. The electrolytes were prepared by dissolving the chosen sodium salt into the specified solvent at a 1 M concentration. The following electrolyte formulations were investigated: sodium hexafluorophosphate (NaPF_6_, purity > 99%, E‐lyte) in diethylene glycol dimethyl ether (2G, Sigma‐ Aldrich), sodium trifluoromethyl sulfonate (NaOTf, purity >98.0 %, TCI) in 2G, NaPF_6_ in ethylene carbonate (EC): diethyl carbonate (DEC) (1:1 vol%), NaOTf in EC:DEC (1:1 vol%), sodium perchlorate (NaClO_4_, Alfa Aesar) in EC: N,N‐dimethylcarbamyl Chloride (DMC, Alfa Aesar): Fluoroethylene carbonate (FEC, Alfa Aesar) (49:49:2 vol%). Residual H_2_O in the solvents was removed by drying the solvents on 4 Å molecular sieves. All electrolytes were prepared and stored in an Ar‐filled glovebox. Sodium foil counter electrodes were made by rolling the sodium lumps into a thin plate, and then using a punching tool to cut into a 12 mm diameter disk. Unless otherwise specified, the mass loading of the active material was ≈1.4 mg cm^−2^, and the area of each electrode was 1.13 cm^2^. For the preparation of the full cells, all the procedures were the same as for the half‐cell assembly. The procedure for making the cathode was the same as the anode, except for the slurry compositions, which were 8:1:1 for the weight of the active material, super C65, and PVDF. Before full‐cell assembly, the SnS/t‐G5 anodes were slightly pre‐sodiated by direct contacting with sodium metal for 5 min in the NaPF_6_/2G electrolyte in order to reduce the capacity loss during the initial cycle.

### Electrochemical Measurements

For all cells assembly took place in an argon‐filled glove box. In two electrodes set up, sodium metal as the counter electrode and SnS/t‐G5 sheet as the working electrode (12 mm diameter). CR2032 coin cells (MIT Corp.) were assembled with the activated electrodes with two glass fiber separators soaked with 100 µL of the electrolyte. In the Swagelok‐type three electrodes cell assembly, using Na as a reference/counter electrode, the 12 mm diameter SnS/t‐G5 electrode was used as the working electrode. Galvanostatic cycling tests were performed with the Biologic BCS 805 test and Neware (CT‐4008) systems in a voltage range between 0.01–2.5 V versus Na^+^/Na at various current densities. For *operando* electrochemical dilatometry experiments, 10 mm diameter electrodes were used as the working electrode, and sodium metal was used as the counter and reference electrodes. EIS characterization was performed with the Biologic BCS 805 test in the frequency range of 1MHz–10m Hz. The DRT data was obtained by using a calculate from the impedance data with open‐source MATLAB R2023b script‐based software (DRT Tools).

### Structural Analysis

*Synchrotron Operando/* ex situ XRD measurements were performed and collected in Debye–Scherrer geometry at beamline P02.1, PETRA III (DESY, Hamburg) at a wavelength of λ = 0.20733 Å (corresponding to an X‐ray energy of ≈60 keV).^[^ ^58^ ^]^ Electrodes for ex situ XRD measurements were extracted from disassembled cells in an Ar‐filled glovebox. The sample was sealed in a 0.5 mm quartz glass capillary and sealed with a wax. 2D data were collected using the Varex XRD 4343CT (150×150 µm2 pixel size, 2880 x 2880 pixel area, CsI scintillator directly deposited on amorphous Si photodiodes). Detector calibration was using the LaB6 SRM 660c standard. Calibration and data integration of 2D images into 1D data were performed using the pyFAI software.^[^ ^59^ ^]^ Rietveld refinement of the powder XRD data was done using GSAS‐II.^[^ ^60^ ^]^ Raman spectroscopy was performed with a Renishaw inVia confocal Raman microscope with a 532 nm laser. To prevent laser damage to the samples, the measurements were performed with 0.1% laser power and an exposure time of 60 s for 3 accumulations. Lab‐based powder for XRD measurements was performed with a Bruker D2 Phaser diffractometer. X‐rays were generated with a Cu Kα source(𝜆 = 1.54056 A) at an operating voltage of 30 kV. Scanning electron microscopy (SEM) was conducted with a Thermo Fisher Scientific Phenom Desktop SEM operating at 15 kV. The microstructures and size were characterized by transmission electron microscopy (TEM, JEM‐2100 F, JEOL, Japan, 200 kV). XPS measurements were performed using a JEOL JPS‐9030 setup, using a non‐monochromated Al‐source (*hν* = 1486.6 eV) for excitation and a pass energy of 10 eV, yielding an energy resolution of 0.96 eV. The binding energy axis was calibrated by measuring Ar^+^ sputter‐cleaned Au and Cu foils and setting the Au 4f_7/2_ peak to 84.00 eV and the Cu 2p_3/2_ peak to 932.66 eV. The three‐electrode ECD cell was assembled in an Ar‐filled glovebox with SnS/t‐G as the working electrode and Na as the reference/counter electrode. The *operando* ECD was measured at a constant temperature of 25 °C using an ECD‐3‐nano device from EL‐CELL, GmbH.

### Computational Details

Calculations based on the following were performed: The interlayer binding energy of SnS was calculated assuming bulk properties, i.e., assuming that the SnS extends infinitely in the c direction, each interlayer binding energy can reflect the real interlayer interaction strength in the bulk phase. For SnS/t‐G model, the AA stacking model of graphite was selected as a reference to simulate the interlayer binding energy after SnS composite graphite, a model was constructed based on the atomic ratio of the composite, in this case containing ≈5 wt.% carbon. Additionally, the model constructed based on the atomic ratio can ensure that the composition of the composite system was reasonably reflected within the calculation range. The interlayer binding energy in the model reflects the binding strength between two layers of graphene and four layers of SnS. This binding energy result was a reasonable approximation of the actual composite system based on the simplification of the model and the limitation of computational power.

All the calculations were performed in the framework of the density functional theory with the projector augmented plane‐wave method, as implemented in the Vienna ab initio simulation package.^[^ ^61^ ^]^ The generalized gradient approximation proposed by Perdew‐Burke‐Ernzerhof (PBE) was selected for the exchange‐correlation potential.^[^ ^62^ ^]^ The cut‐off energy for the plane wave was set to 480 eV. The energy criterion was set to 10^−4^ eV in the iterative solution of the Kohn‐Sham equation. All the structures were relaxed until the residual forces on the atoms were declined to less than 0.05 eV Å^−1^. To avoid interlaminar interactions, a vacuum spacing of 20 Å was applied perpendicular to the slab.

The combination energies (*E*_com_) were calculated as follows:

(3) $$\Delta E_{\mathrm{com}}=E_{A}+B-E_{A}-E_{B}$$

where *E*_A+B_ was the total energy of the slab A model with B adsorption, *E*_A_ was the energy of a A slab, and *E*_B_ was that for a B molecule.

## Conflict of Interest

The authors declare no conflict of interest.

## Supporting information



Supporting Information

## Data Availability

The data that support the findings of this study are available from the corresponding author upon reasonable request.
