# Supplementary material for: SnS Anodes with High Volumetric Capacity for Na‐ion Batteries and Their Characterization in Ether and Ester Electrolytes
Source: Small. 2025 Aug 26;21(40):e03066. doi: 10.1002/smll.202503066 (PMC12508710; doi:10.1002/smll.202503066)
Supplement: Supplementary file 1 — Supporting Information [file SMLL-21-e03066-s001.docx]

**Supporting information**

**for**

**SnS anodes with high volumetric capacity for Na-ion batteries** **and their characterization in ether and ester electrolytes**

Hui Wang^1^, Yanan Sun^*1,2^, Thorsten Schultz^3,5^, Katherine A. Mazzio^1,2^, Vinita Ahuja^1^, Yongchun Li^1^, Volodymyr Baran^4^, Norbert Koch^3,5,6^, Philipp Adelhelm^*1,2,6^

^1:^ Department of Chemistry, Humboldt University of Berlin, Brook-Taylor-Str. 2,12489 Berlin, Germany

^2^ Joint research group CE-GOBA, Helmholtz-Zentrum Berlin für Materialien und Energie (HZB), 12489 Berlin, Germany

^3:^ Joint research group SE-GHM, Helmholtz-Zentrum Berlin für Materialien und Energie (HZB), Hahn-Meitner-Platz 1, 14109 Berlin, Germany

^4:^ Deutsches Elektronen-Synchrotron (DESY), Notkestraße 85, 22607 Hamburg, Germany

^5:^ Department of Physics, Humboldt University of Berlin, Zum Großen Windkanal 2, 12489 Berlin, Germany

^6:^ Center for the Science of Materials Berlin (CSMB), Humboldt University of Berlin, Zum Großen Windkanal 2, 12489 Berlin, Germany

^*:^ Corresponding author email: philipp.adelhelm@hu-berlin.de (Philipp Adelhelm), yanan.sun@helmholtz-berlin.de (Yanan Sun)

**Supplementary Figures**





**Figure S1.** X-ray diffraction (XRD) patterns of Sn+S powder by hand mixing. No indication of SnS can be found. (Cu K𝛼 radiation, 𝜆 = 1.54056 Å)





**Figure S2.** XRD patterns of Sn+S powder through different mill speeds. (Cu K𝛼 radiation, 𝜆 = 1.54056 Å)





**Figure S3.** XRD diffraction and Rietveld refinement of pure SnS. The solid circle represents the Sn_2_S_3_ impurity.


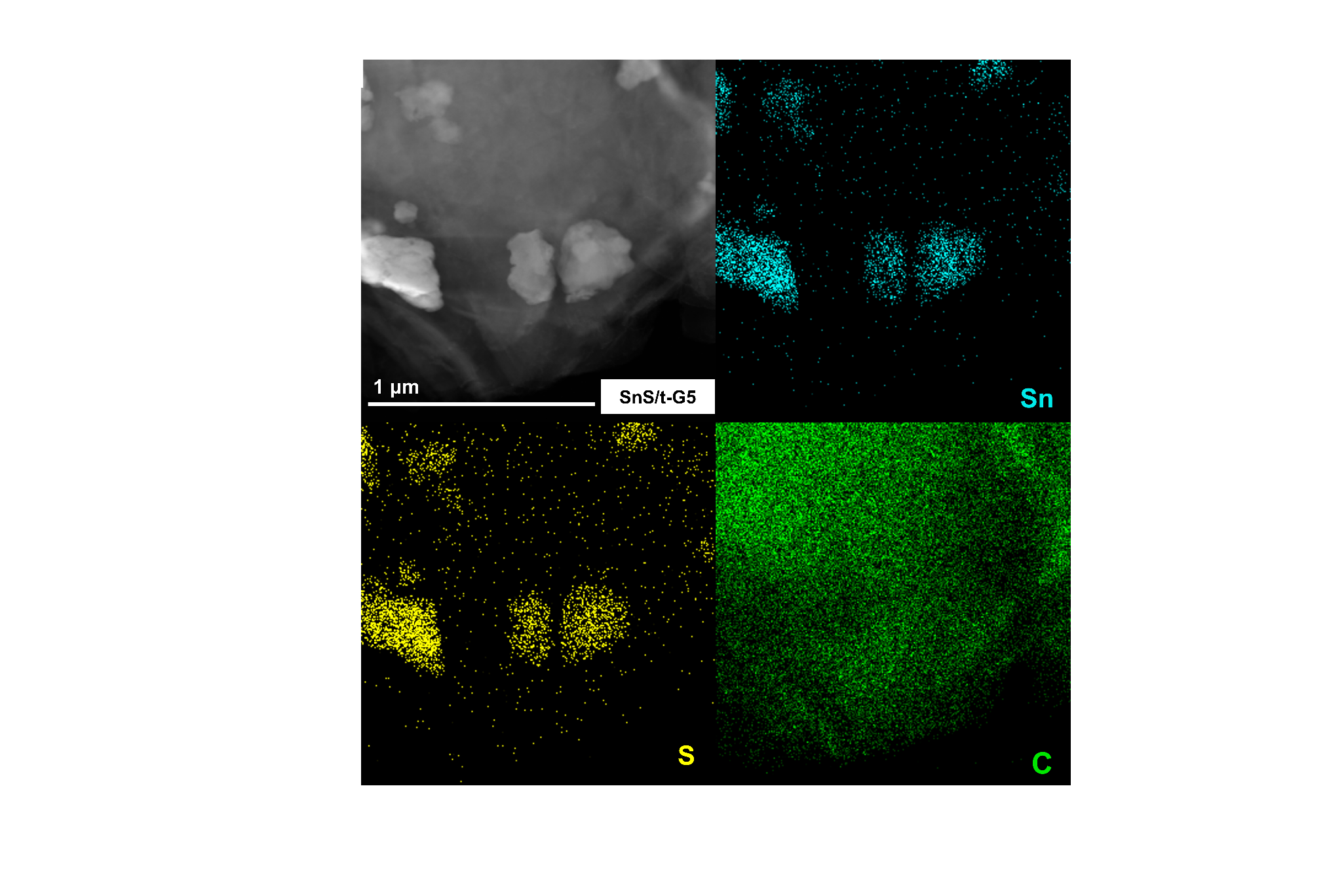


**Figure S4.** TEM image of SnS/t-G5 and the corresponding elemental mapping images (Sn, S, and C).


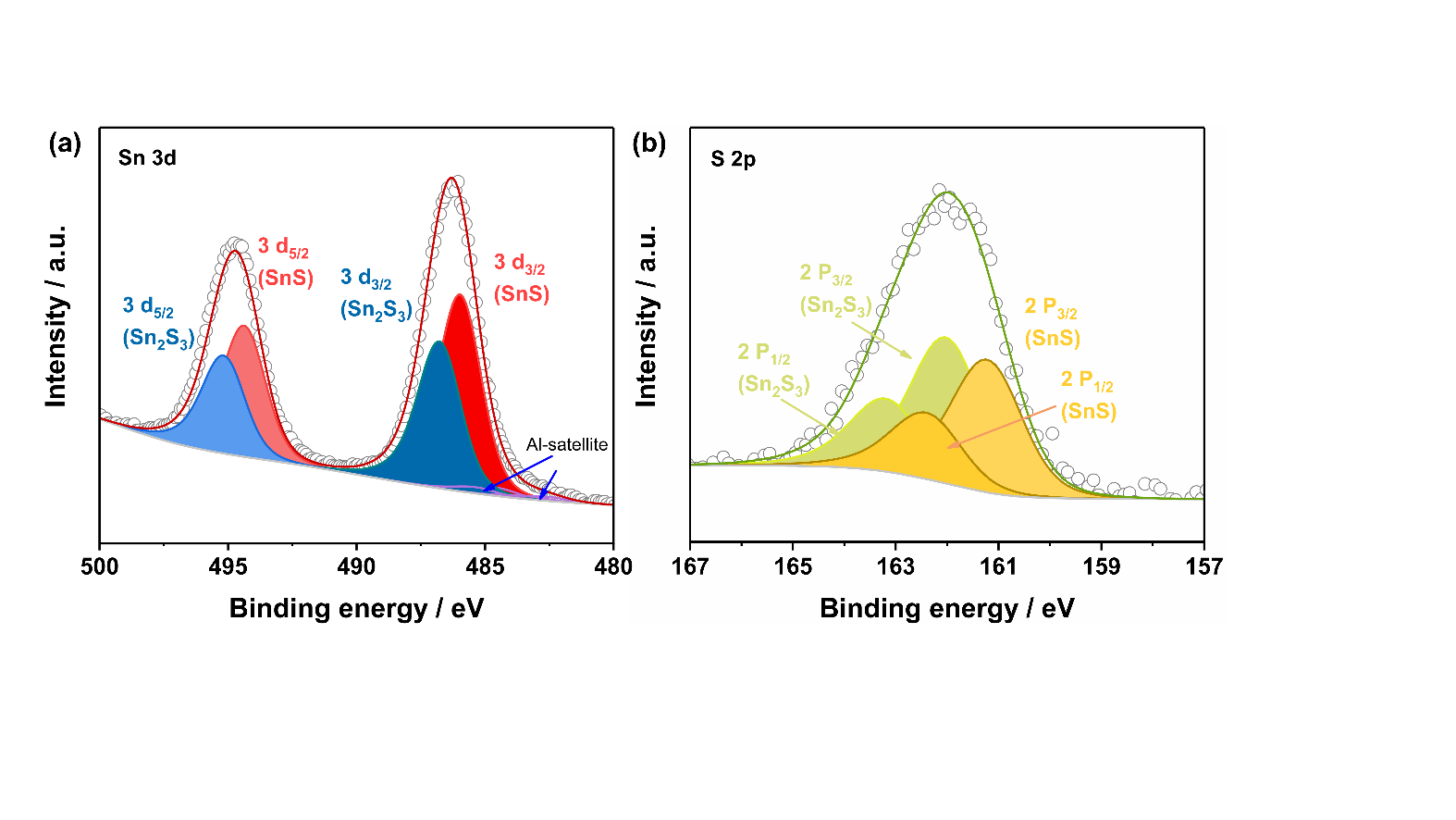


**Figure S5.** The XPS spectra (a) Sn 3d and (b) S 2p of pure SnS.





**Figure S6.** The second galvanostatic charge/discharge curves of SnS/t-G (0, 5, 10, 20, 50 and 85 wt.%) electrodes at a current of 100 mA g^−1^ in a voltage window of 0.01–2.5 V *vs.* Na^+^/Na.

**

**

**Figure S7.** The Coulomb efficiency of SnS/t-G (0 wt.%, 5 wt.%, 10 wt.%, 20 wt.%, 50 wt.% and 85 wt.%) and t-G electrodes at a current density of 1.0 A g^−1^ in a voltage window of 0.01–2.5 V *vs.* Na^+^/Na.

**
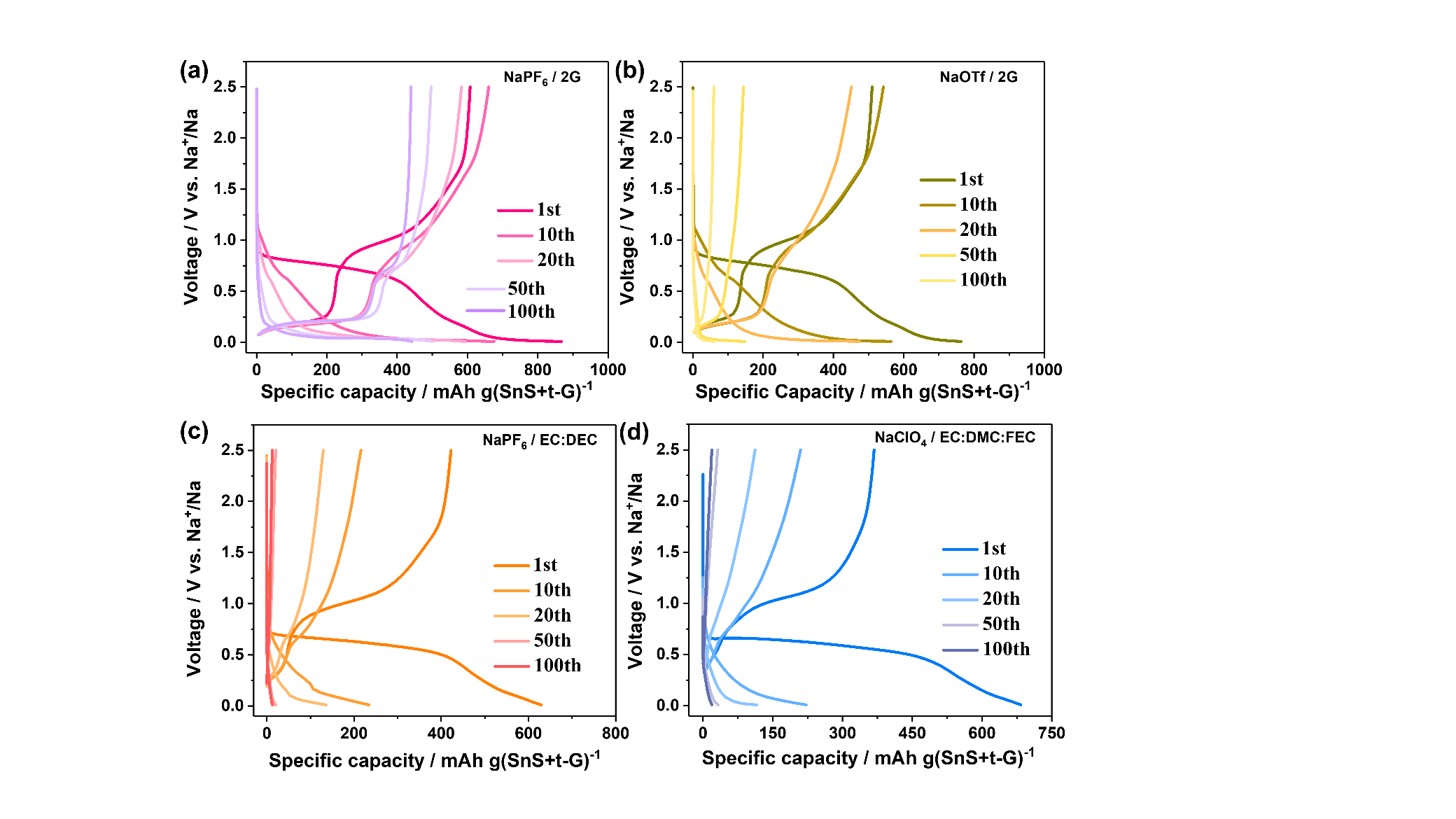
**

**Figure S8.** Galvanostatic discharge/charge profiles of SnS/t-G5 electrodes in an electrolyte comprising (a) NaPF_6_/2G, (b) NaOTf/2G, (c) NaPF_6_/EC:DEC, and (d) NaClO_4_/EC:DMC:FEC electrolyte for 1, 10, 20, 50, and 100 cycles at a current density of 1.0 A g^−1^ in a voltage window of 0.01–2.5 V *vs.* Na^+^/Na. NaOTf/EC:DEC electrolyte was unable to cycling within the SnS/t-G5 system under high current density.





**Figure S9.** Differential capacity *vs.* voltage curves of SnS/t-G5 electrode at the first cycle in different electrolytes at a current density of 100 mA g^−1^ in a voltage window of 0.01–2.5 V *vs.* Na^+^/Na.


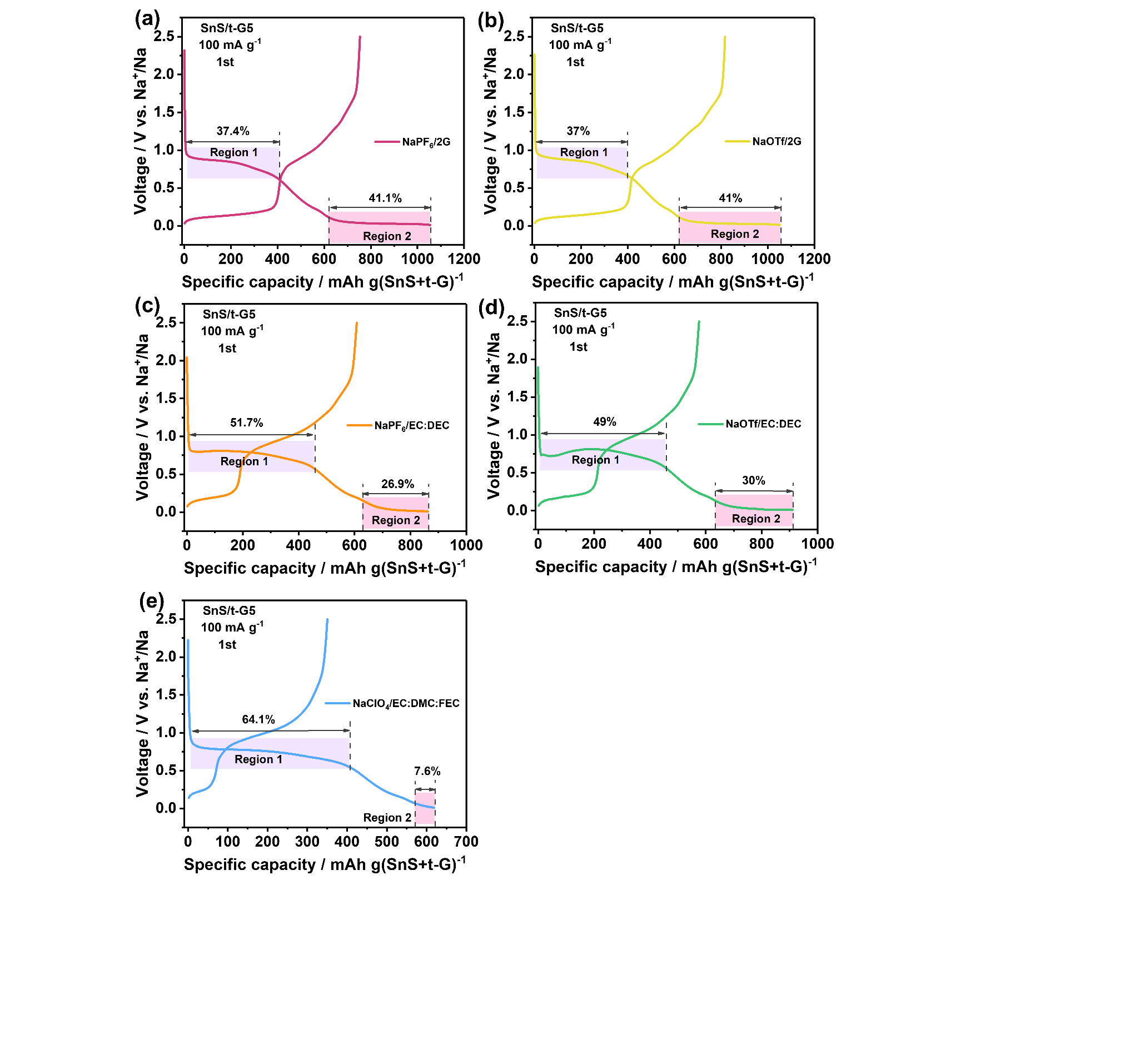


**Figure S10.** The SnS/t-G5 electrode at first cycle in different electrolytes at 100 mA g^-1^ in a voltage window of 0.01–2.5 V *vs.* Na^+^/Na. (a) NaPF_6_/2G. (b) NaOTf /2G. (c) NaPF_6_/EC:DEC. (d) NaOTf/EC:DEC. (e) NaClO_4/_EC:DMC:FEC.

.


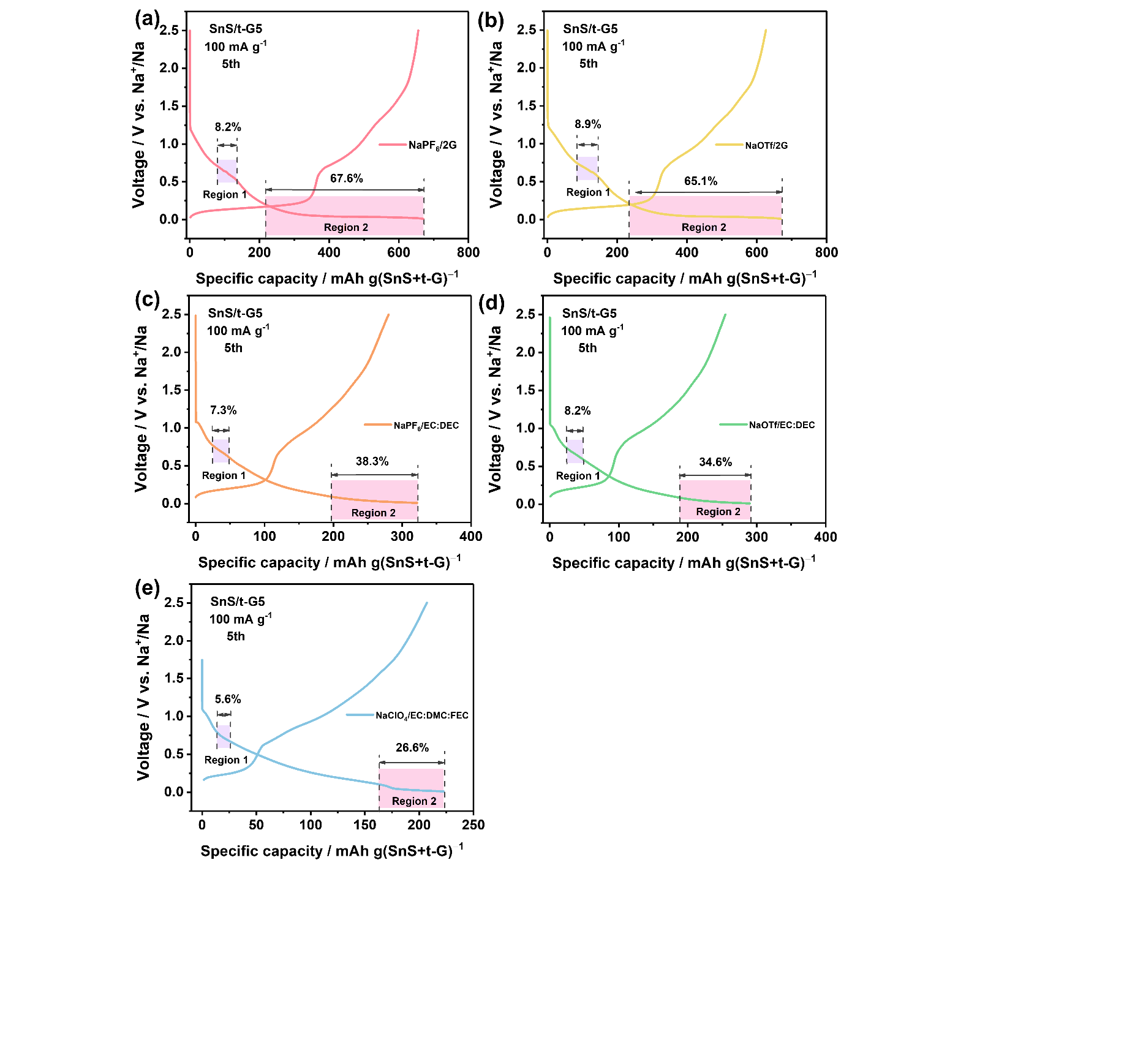


**Figure S11.** The SnS/t-G5 electrode at fifth cycles in different electrolytes at 100 mA g^-1^ in a voltage window of 0.01–2.5 V *vs.* Na^+^/Na. (a) NaPF_6_/2G. (b) NaOTf /2G. (c) NaPF_6_/EC:DEC. (d) NaOTf/EC:DEC. (e) NaClO_4_/EC:DMC:FEC.


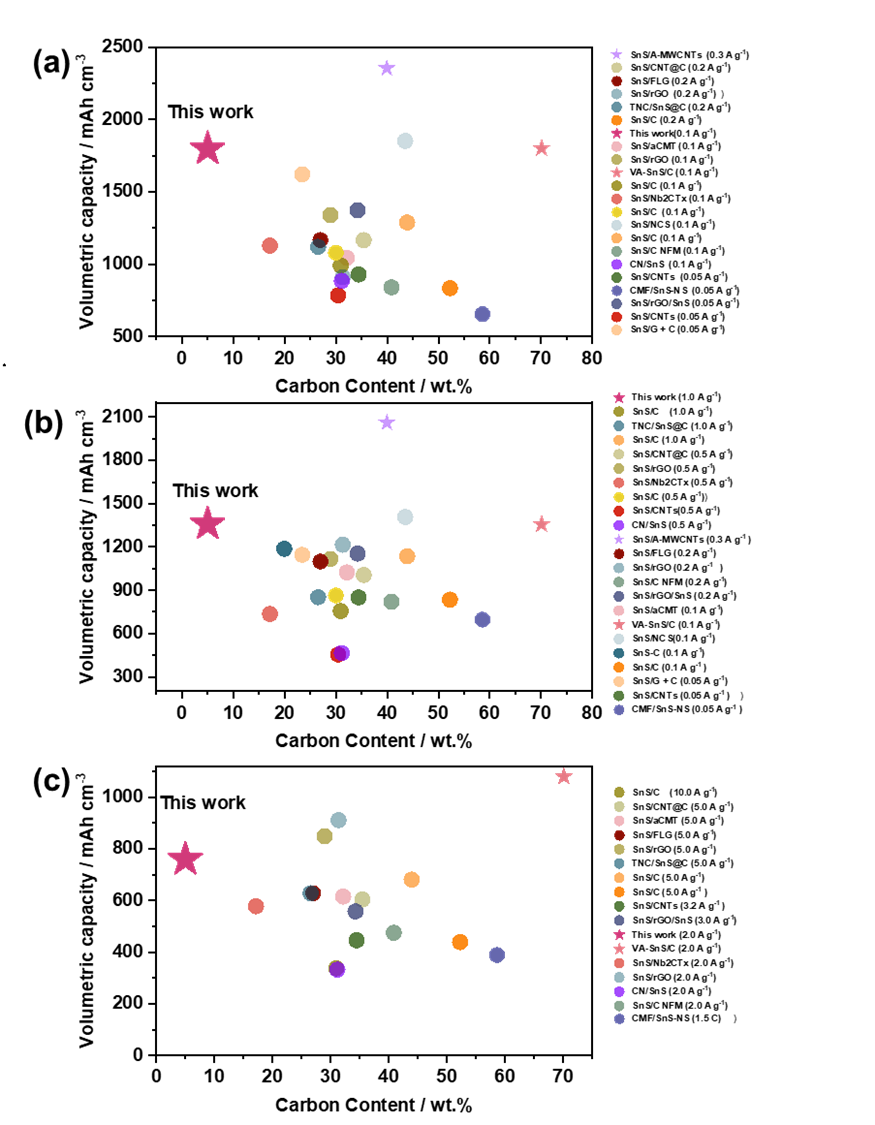


**Figure S12**. Comparison of volumetric capacity (based on sodiated SnS) of SnS/carbon electrodes in the (a) 2nd cycle, (b) 25th cycle (cycling stability) and (c) at high current density (rate performance). The corresponding current densities are indicated in the figure. The use of ether-based electrolytes and ester-based electrolytes in different material systems is indicated by solid stars and circles, respectively.


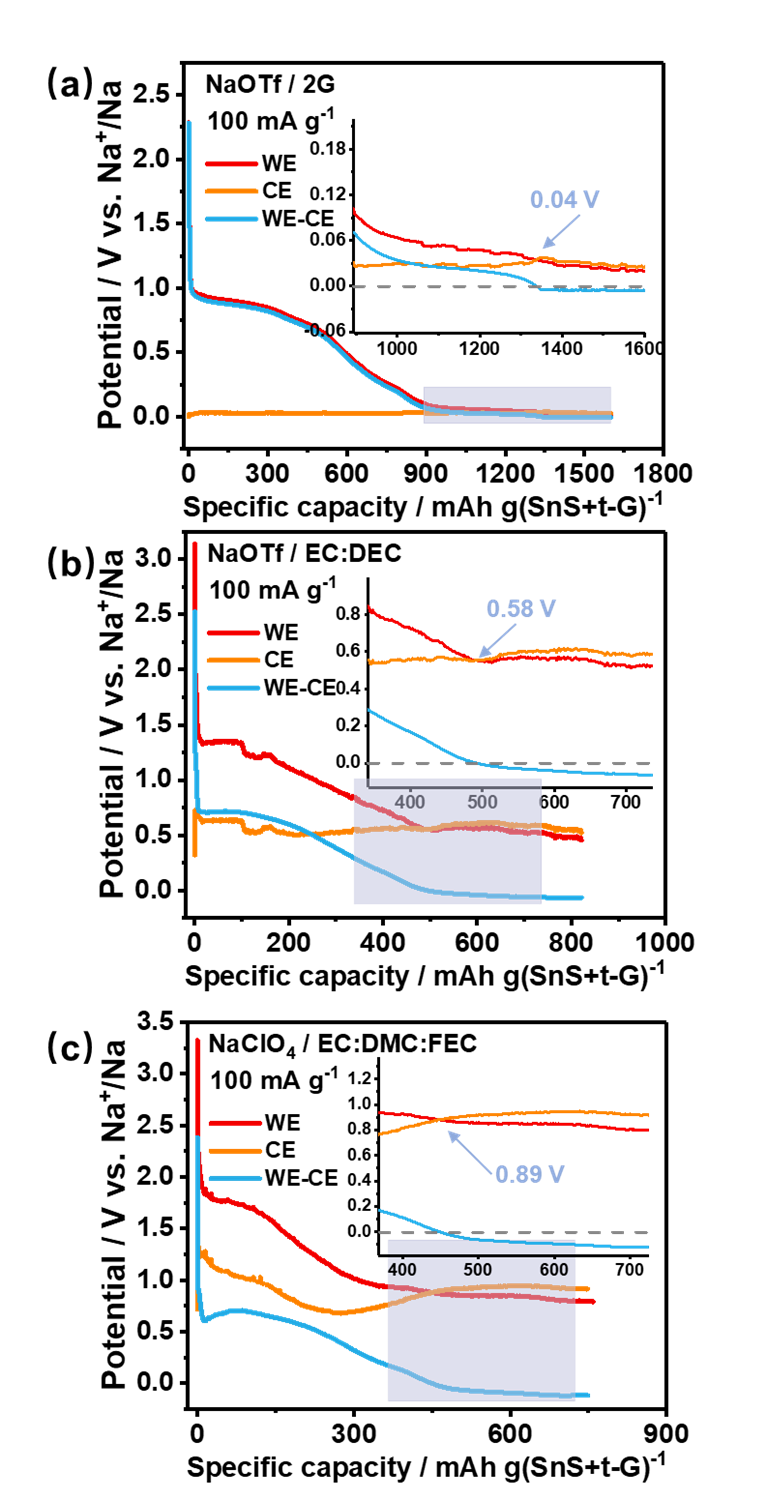


**Figure S13**. Initial discharge curves of SnS/t-G5 electrode at 100 mA g^−1^ in the three-electrode Swagelok-type cell when using the (a) NaOTf/2G. (b) NaOTf/EC:DEC. (d) NaClO_4_/EC:DMC:FEC.


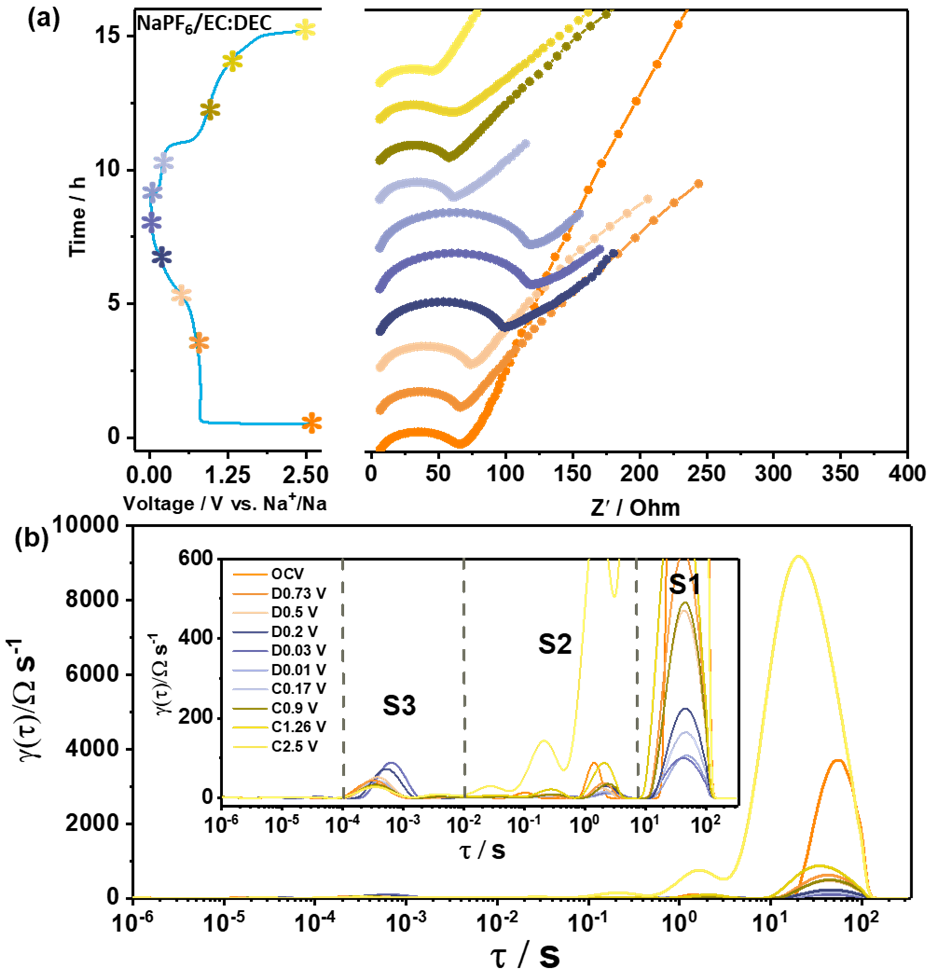


**Figure S14.** (a)The *in‐situ* EIS spectra in NaPF_6_/EC:DEC. The initial cycle discharge and charge profile are plotted at the left side of each spectrum. (b) DRT curves from *in-situ* EIS measurements at different potentials.


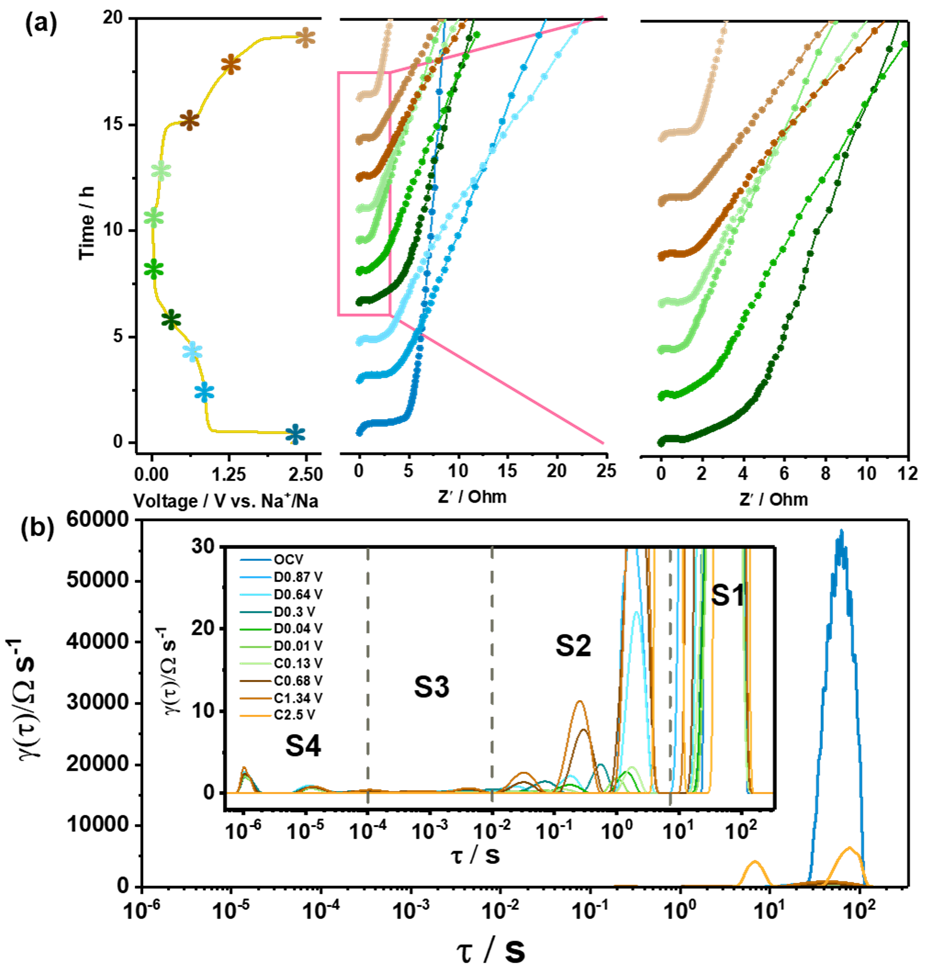


**Figure S15**. (a)The *in‐situ* EIS spectra in NaOTf/2G. The initial cycle discharge and charge profile are plotted at left side of each spectrum. (b) DRT curves from *in-situ* EIS measurements at different potentials.


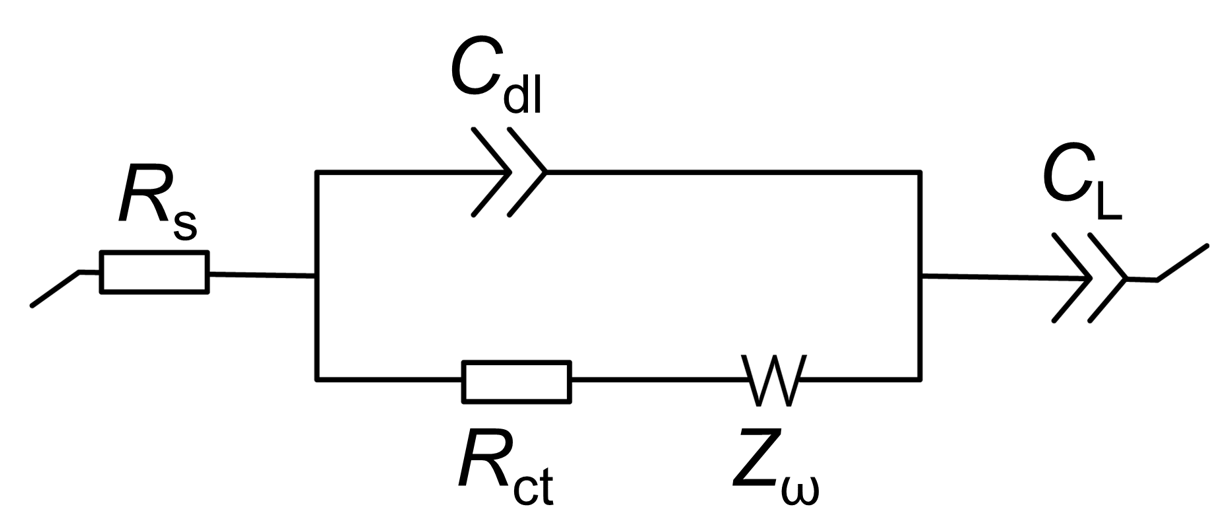


**Figure S16.** Equivalent circuit model for EIS analysis.

**

**

**Figure S17.** XRD pattern of the Na_3_V_3_(PO_4_)_3_/C powder. (Cu K𝛼 radiation, 𝜆 = 1.54056 A)





**Figure S18.** Cycling performance of SnS/t-G5 // NVP/C full cell at 100 mA g^−1^ in different electrolytes.


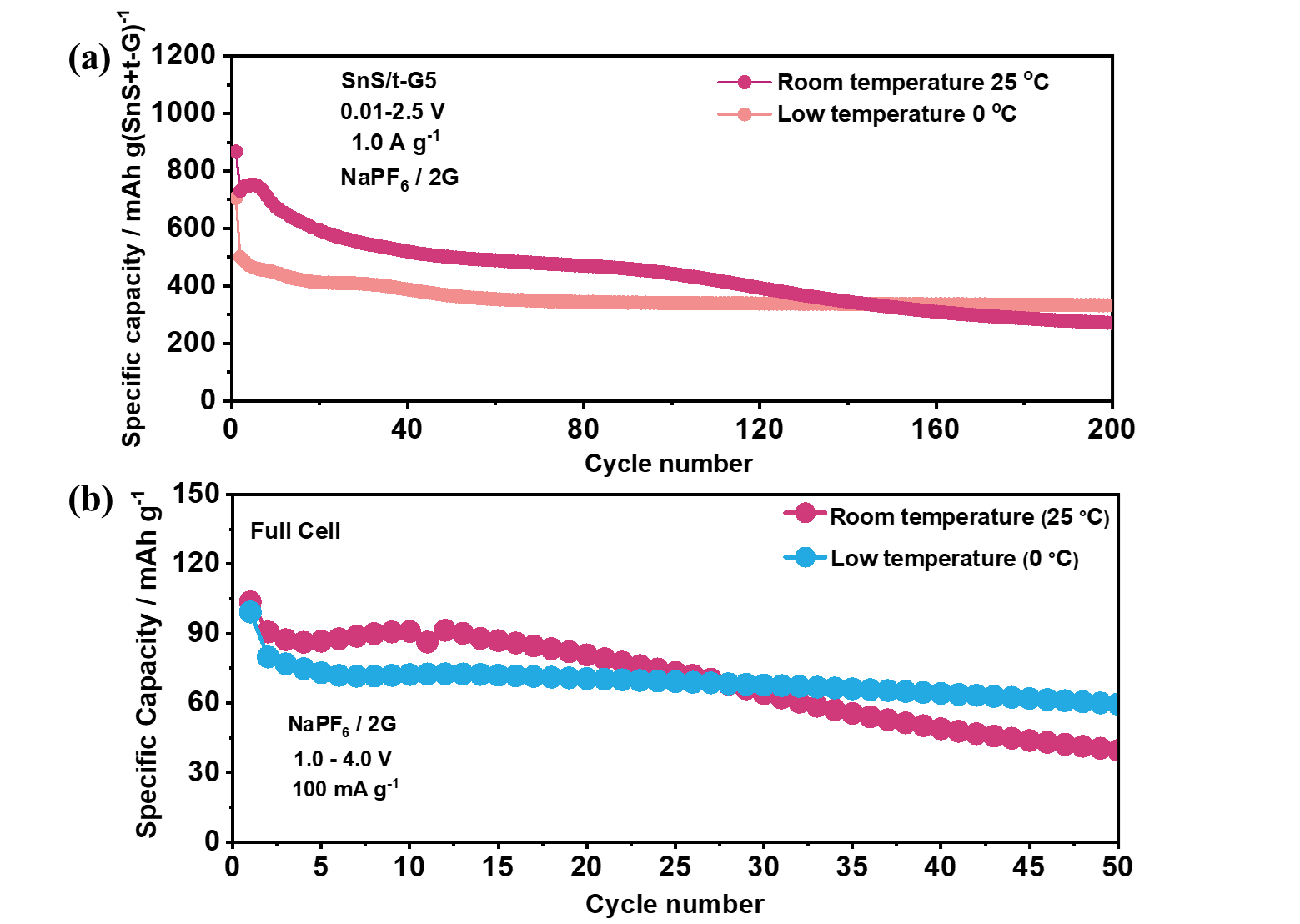


**Figure S19.** Cycling performance of SnS/t-G5 electrode in both half-cell and full-cell configurations at room temperature and low temperature.

**Calculations on theoretical capacity and volume expansion**

**a) Calculations based on the bulk (theoretical) properties of the materials:**

For conversion reactions, values for the theoretical capacities per volume (mAh cc^−1^) can be very different depending on whether the sodiated or the non-sodiated state is considered. This is because the different phases formed during the reaction have very different densities, leading to large volume changes during charge/discharge.

The values stated in the manuscript are calculated using the values and formulas summarized below. Theoretical capacity per weight (mAh g^−1^) values are also given with and without the weight of sodium. As SnS is a negative electrode, the weight of Na can be neglected (just as when calculating e.g. the theoretical capacity of hard carbon or any other negative electrode).

|  | **Molar mass / g mol**^−1^ | **Density / g cc**^−1^ | **Molar volume / cc mol**^−1^ |
| --- | --- | --- | --- |
| **Na** | 22.99 | 0.97 | 23.70 |
| **Sn** | 118.71 | 7.29 | 16.28 |
| **S** | 32.07 | 2.07 | 15.49 |
| **SnS** | 150.78 | 5.22 | 28.89 |
| **Sn_3.75_Sn** | 204.92 | 2.40 | 85.38 |
| **Na_2_S** | 78.05 | 1.90 | 41.08 |

Formulas for the calculation of theoretical capacities

Theoretical gravimetric capacity *q*_th,grav_:

$$\boldsymbol{q}_{\boldsymbol{th, grav}}\boldsymbol{[mAh}\boldsymbol{g}^{\boldsymbol{-1}}\boldsymbol{]=}\frac{\boldsymbol{x\times F}}{\boldsymbol{3.6\times M}}$$

Theoretical volumetric capacity *q_th,vol_*:

$$\boldsymbol{q}_{\boldsymbol{th, vol}}\boldsymbol{[mAh}\boldsymbol{cc}^{\boldsymbol{-1}}\boldsymbol{]=}\frac{\boldsymbol{x\times F}}{\boldsymbol{3.6\times}\boldsymbol{V}_{\boldsymbol{m}}}$$

With *F* being the Faraday constant and *M* and *V_m_* being the molar mass or molar volume of either SnS (before sodiation) or Na_3.75_Sn+Na_2_S (after sodiation). *Δx* is the amount of Na stored per SnS, i.e. 5.75.

The total reaction (3) consists of the two steps (1) and (2)

Conversion reaction: SnS + 2Na^+^ + 2e^-^ ⇌ Sn + Na_2_S (1)

Alloying reaction: Sn + 3.75Na^+^ + 3.75e^-^ ⇌ Na_3.75_Sn (2)

Total reaction: 5.75 Na + SnS ⇌ Na_3.75_Sn + Na_2_S (3)

For the total reaction, the theoretical values are calculated as:

before sodiation: *q_th,grav_*= 1022.07 mAh g^−1^ and *q_th,vol_*= 5335.21 mAh cc^−1^, and

after sodiation: *q_th,grav_*= 544.60 mAh g^−1^ and *q_th,vol_*= 1218.60 mAh cc^−1^

The volume expansion during sodiation is calculated from the molar volumes of SnS, Na_3.75_Sn and Na_2_S.

$$Expansion \left[ \% \right]=\left[ \frac{\left( V_{m,sodiated} \right)-V_{m,non-sodiated}}{V_{m,non-sodiated}} \right]\times100\%=\left[ \frac{\left( V_{m,Na3.7Sn}+V_{m,Na2S} \right)-V_{m,SnS}}{V_{m,SnS}} \right]\times100\%$$

For the formation of Na_3.75_Sn and Na_2_S from SnS, the volume expansion amounts to 338 %.

**b) Volumetric capacity calculations of composites:**

Formulas for the calculation of volumetric capacities of composites:

$$\boldsymbol{q}_{\boldsymbol{com, vol}} \left[ \boldsymbol{mAh}\boldsymbol{cc}^{\boldsymbol{-1}} \right]\boldsymbol{=}\boldsymbol{q}_{\boldsymbol{com,grav}}\boldsymbol{\times}\boldsymbol{\rho}_{\boldsymbol{com}}$$

$\boldsymbol{=}\boldsymbol{q}_{\boldsymbol{com,grav}}\boldsymbol{\times(}\boldsymbol{\omega}_{\boldsymbol{A}}\boldsymbol{\times}\boldsymbol{\rho}_{\boldsymbol{A}}\boldsymbol{+}\boldsymbol{\omega}_{\boldsymbol{B}}\boldsymbol{\times}\boldsymbol{\rho}_{\boldsymbol{B}}\mathbf{)}$

With *q_com,_*_grav_ being the gravimetric capacity obtained from the experiment, and *ρ*_com_ and *ω*_com_ being the density and mass fraction of the composite material. *ρ_A_* and *ω_A_* are the density and mass fraction of SnS, respectively, while *ρ_B_* and *ω_B_* are the density and mass fraction of graphite, respectively.

The volumetric capacity is calculated as:

|  | **Gavimetric capacity/mAh g**^−1^ **(Measured value)** | **Weight fraction of SnS (*ω* SnS)** | **Weight fraction of graphite (*ω* graphite)** | **Composite density/ g cc**^−1^ |
| --- | --- | --- | --- | --- |
| **SnS** | 1099 | 1.00 | 0.00 | 5.22 |
| **SnS/t-G5** | 1055 | 0.95 | 0.05 | 5.07 |
| **SnS/t-G10** | 1010 | 0.90 | 0.10 | 4.93 |
| **SnS/t-G20** | 863 | 0.80 | 0.20 | 4.63 |
| **SnS/t-G50** | 577 | 0.50 | 0.50 | 3.75 |
| **SnS/t-G85** | 368 | 0.15 | 0.85 | 2.71 |
|  | | | | |
| **SnS density / g cc**^−1^ | **graphite density / g cc**^−1^ | $\boldsymbol{\rho}_{\boldsymbol{com}}$**=** $\boldsymbol{\omega}_{\boldsymbol{SnS}}\boldsymbol{\times}\boldsymbol{\rho}_{\boldsymbol{SnS}}\boldsymbol{+}\boldsymbol{\omega}_{\boldsymbol{graphite}}\boldsymbol{\times}\boldsymbol{\rho}_{\boldsymbol{graphite}}$ | | |
| 5.22 | 2.27 |  |  |  |

| **Volumetric capacity of the composites** | | |
| --- | --- | --- |
| *q* vol(SnS)= | 5736.78 | mAh cc^−1^ |
| *q* vol(SnS/t-G5)= | 5351.49 | mAh cc^−1^ |
| *q* vol(SnS/t-G10)= | 4974.25 | mAh cc^−1^ |
| *q* vol(SnS/t-G20)= | 3995.69 | mAh cc^−1^ |
| *q* vol(SnS/t-G50)= | 2160.87 | mAh cc^−1^ |
| *q* vol(SnS/t-G85)= | 998.20 | mAh cc^−1^ |

.

**c) Volumetric capacity calculations of electrodes:**

The effective volumetric capacity of an electrode depends on the volumetric capacity of the materials as well as the electrode porosity. The electrode porosity can be calculated from the composition of the electrode (contents of active material, additive and binder), the loading (mg cm^−2^) and the thickness (µm) of the electrode. At the same time, the volumetric capacity of commercial hard carbon electrode^[1]^ was calculated for comparison.

(1) Formulas for the calculation of volumetric capacities of electrodes:

$$\boldsymbol{q}_{\boldsymbol{elec,vol}} \left[ \boldsymbol{mAh}\boldsymbol{cc}^{\boldsymbol{-1}} \right]\boldsymbol{=L\times h}$$

Where *L* is the area capacity of either $q_{com,grav}\times\frac{m_{com}}{A_{elec}}$(SnS/t-G5 electrode) or $q_{HC,grav}\times coating weigh \times0.9$(hard carbon), and *h* is thickness of electrode (w/o current collector). The capacity of hard carbon ($q_{HC,grav}$) is assumed as 300 mAh g^−1^, while the weight fraction of hard carbon ($w_{HC,grav}$) in a commercial electrode is assumed as 0.9.

The volumetric capacity is calculated as:

|  | **SnS/t-G5 electrode** | **Hard carbon electrode (double coated)** |
| --- | --- | --- |
| **Area of electrode (*A*) / cm^2^** | 1.13 | 857.86 |
| **Thickness of electrode (*h*) (w/o current collector) / cm** | 0.0036 | 0.0154 |
| **Gravimetric capacity (*q_grav_*) / mAh g**^−1^ | 1055.00 | 300.00 |
| **Mass of active material (*m*) / g** | 0.00109 | 13.70 |
| **Area capacity (*L*) / mAh cm**^−^**^2^** | 1.02 | 4.33 |
| **Volumetric capacity (*q_vol_*) / mAh cc**^−1^ | 282.68 | 280.87 |

(2) Calculating porosity of SnS/t-G5 and hard carbon electrodes:

| **Parameter** | **Symbol** | **SnS/t-G5** | **Hard carbon** |
| --- | --- | --- | --- |
| **Electrode Thickness excluding current collector (cm)** | *h* | 0.0036 | 0.0154 |
| **Electrode Area (cm^2^)** | *A* | 1.13 | 857.86 |
| **Active Material Mass (g)** | *m(active)* | 0.00109 | 13.74 |
| **Active material Mass Ratio** | *w(active)* | 0.7 | 0.9 |
| **Additive Mass Ratio** | *w(additive)* | 0.2 | 0.05 |
| **Binder Mass Ratio** | *w(binder)* | 0.1 | 0.05 |
| **Total Electrode Mass (g)** | *m(electrode)* = *m(active)* / *w(active)* | 0.00156 | 15.27 |
| **Additive Mass (g)** | *m(additive)* = *m(electrode)* $\times$ *w(additive)* | 0.00031 | 0.76 |
| **Binder Mass (g)** | *m(binder)* = *m(electrode)* $\times$ *w(binder)* | 0.00016 | 0.76 |
| **Active Density (g cc**^−1^**)** | *ρ(active)* | 4.9 | 1.6 |
| **Additive Density (g cc**^−1^**)** | *ρ(additive)* | 1.6 | 1.6 |
| **Binder Density (g cc**^−1^**)** | *ρ(binder)* | 1.78 | 1.78 |

*: The *w(active),* *w(additive) and w(binder)* of hard carbon are assumed as 0.9, 0.05, and 0.05, respectively. While the *ρ(active)* of hard carbon is assumed as 1.6 g cc^−1^.

The porosity is calculated as:

| **Steps to Calculate Porosity and Use it to Determine Volumetric Capacity** | **Formula** | **SnS/t-G5** | **Hard carbon** |
| --- | --- | --- | --- |
| **Step 1: Calculate Total Electrode Volume (cc)** | *V (total, electrode)* = *h* $\times$ *A* | 0.004068 | 13.21 |
|  | | | |
| **Step 2: Calculate the Volume of Each Component (cc)** | *V (active)* = *m (active)* / *ρ (active)* | 0.000222 | 8.59 |
|  | *V (additive)* = *m (additive)* / *ρ (additive)* | 0.000195 | 0.48 |
|  | *V (binder)* = *m (binder)* / *ρ (binder)* | 0.000087 | 0.43 |
|  | *V (total, material)* = *V (active)* + *V (additive)* + *V (binder)* | 0.000505 | 9.50 |
|  | | | |
| **Step 3: Calculate Porosity (%)** | *ε* = 1 - (*V (total, material)* / *V* *(total, electrode)*) | 87.6% | 28.1% |

**Supplementary tables**

**Table S1.** SnS and graphite crystallographic parameters of the SnS/t-G5 composite by Rietveld refinement.

| SnS site | *x* | | | *y* | | *z* | | Wyckoff symbol | | Occ. |
| --- | --- | --- | --- | --- | --- | --- | --- | --- | --- | --- |
| Sn^2+^ | 0.1185 | | | 0.25 | | 0.1213 | | 4c | | 1 |
| S | 0.1428 | | | 0.75 | | 0.5176 | | 4c | | 1 |
| Space group: P n m a ; a =11.20132 Å b = 3.98537 Å c= 4.3293 Å; | | | | | | | | | | |
| Graphite site | *x* | | *y* | | *z* | | Wyckoff symbol | | Occ. | |
| C1 | 0.0 | | 0.0 | | 0.25 | | 2b | | 1 | |
| C2 | 0.3333 | | 0.6667 | | 0.25 | | 2c | | 1 | |
| Space group: P 63/m m c ; a =1.9685Å b = 1.9685Å c= 6.7028Å | | | | | | | | | | |
| Refinement results | | *Rwp* = 4.982% | | | | | | | | |

**Table S2.** Crystallographic parameters of the SnS by Rietveld refinement.

| Site | *x* | *y* | *z* | Wyckoff symbol | Occ. |
| --- | --- | --- | --- | --- | --- |
| Sn^2+^ | 0.11939 | 0.25 | 0.12131 | 4c | 1 |
| S | 0.1477 | 0.75 | 0.5176 | 4c | 1 |
| Space group: P n m a ; a =11.19548Å b = 3.98421Å c= 4.32916Å | | | | | |
| Refinement results | *Rwp* = 6.063% | | | | |

**Table S3.** Details of interlayer binding energy

| **Model** | ***E*_A+B_ (eV)** | ***E*_A_ (eV)** | ***E*_B_ (eV)** | ***E*_com_ (eV)** |
| --- | --- | --- | --- | --- |
| SnS | -36.947 | -17.968 | -17.968 | -1.010 |
| SnS/t-G | -368.136 | -143.997 | -221.853 | -2.285 |

**Table S4.** Details of electrolytes used in SnS/t-G5 electrode.

| **Electrolyte** | | **Concentration** | **Salt** | **Solvent** | **Additive** |
| --- | --- | --- | --- | --- | --- |
| **Ether-based** | 1M NaPF­­_6_ in 2G | 1 mol/L | NaPF­­_6_ | 2G |  |
|  | 1M NaOTf in 2G | 1 mol/L | NaOTf | 2G |  |
| **Ester-based** | 1M NaPF­­_6_ in EC:DEC | 1 mol/L | NaPF_6_ | EC + DEC (50:50 vol%) |  |
|  | 1M NaOTf in EC:DEC | 1 mol/L | NaOTf | EC + DEC (50:50 vol%) |  |
|  | 1M NaClO_4_ in EC:DMC:FEC | 1 mol/L | NaClO_4_ | EC + DMC (49:49 vol%) | FEC  (2 vol%) |

| **Table S5.** A comparison of the electrochemical performance of some representative SnS/carbon composites in SIBs. | | | | | | | | | | | | |
| --- | --- | --- | --- | --- | --- | --- | --- | --- | --- | --- | --- | --- |
| **Materials** | **Carbon**  **source** | **Carbon content**  **(wt%)** | **Electrode Formulation**  **(wt%)** | **Electrolyte**  **(1M)** | **ICE**  **(%)** | **1^st^ discharge**  **capacity**  **/mAh g(electrode)^-1^** | **1^st^ Volumetric capacity**  **/mAh cc(un-sodiated)^-1^** | **Mass**  **loading**  **(mg cm^-2^)** | **2^nd^ discharge**  **capacity**  **/mAh g(electrode)^-1^** | **Current density**  **/ A g^-1^** | **Voltage range**  **/ V** | **Ref.** |
|  |  |  |  |  |  |  | **1^st^ Volumetric capacity**  **/mAh cc(full-sodiated)^-1^** |  |  |  |  |  |
| **SnS/t-G5** | **Thermally activated graphite** | **5.0** | **7:2:1(AM : CA : PVDF)** | **NaPF_6_/ 2G** | **71.4** | **1055** | **5351** | **1.4** | **751** | **0.1** | **0.01-2.5** | **This work** |
|  |  |  |  |  |  |  | **2385** |  |  |  |  |  |
| SnS/CNT@C | Carbon nanotubes, C | 35.5 | 7:1.5:1.5(AM : CA : CMC） | NaClO_4_ /EC:PC:FEC | 71.1 | 819 | 3135 | 0.6 | ~580 | 0.2 | 0.01-3.0 | ^[2]^ |
|  |  |  |  |  |  |  | 1572 |  |  |  |  |  |
| SnS/aCMT | Porous carbon microtubes | 32.2 | 8:1:1(AM : CA : PVDF） | NaClO_4_/EC:DMC:FEC | 76.0 | 547 | 2165 | 1.3 | Not mentioned | 0.5 | 0.01-3.0 | ^[3]^ |
|  |  |  |  |  |  |  | 1067 |  |  |  |  |  |
| SnS/rGO | S and N Co-doped Graphene | 29.0 | 8:1:1(AM : CA : CMC) | NaClO_4_/PC:FEC | 74.0 | 807 | 3417 | 1.1 | ~600 | 0.1 | 0.01-2.5 | ^[4]^ |
|  |  |  |  |  |  |  | 1721 |  |  |  |  |  |
| VA-SnS/C | Carbon | 70.1 | AM | NaPF_6_/2G | 89.8 | 858 | 2704 | 0.3 | ~800 | 0.1 | 0.01-2.0 | ^[5]^ |
|  |  |  |  |  |  |  | 1933 |  |  |  |  |  |
| SnS/C | Carbon | 31.0 | AM | NaClO_4_/PC:FEC | 79.0 | 523 | 2291 | N/A | ~420 | 0.1 | 0.01-3.0 | ^[6]^ |
|  |  |  |  |  |  |  | 1204 |  |  |  |  |  |
| SnS/Nb_2_CTx | Nb_2_CT_x_ Mxene | 17.2 | 8:1:1(AM: CA : CMC) | NaClO_4_/EC:PC:FEC | 54.9 | 744 | 3689 | 1.5 | ~430 | 0.1 | 0.01-3.0 | ^[7]^ |
|  |  |  |  |  |  |  | 1866 |  |  |  |  |  |
| SnS/C | Super P carbon | 30.0 | 7:1.5:1.5(AM : CA : PAA) | NaClO_4_/EC:PC:FEC | ~81.0 | ~ 610 | 2522 | 1.4 | ~500 | 0.1 | 0.01-3.0 | ^[8]^ |
|  |  |  |  |  |  |  | 1258 |  |  |  |  |  |
| SnS/CNTs | Carbon nanotube | 30.5 | 8:1:1(AM : CA : PVDF) | NaClO_4_/EC:DMC | 41.7 | 833 | 3352 | 1.0 | ~380 | 0.05 | 0.01-2.0 | ^[9]^ |
|  |  |  |  |  |  |  | 1639 |  |  |  |  |  |
| SnS/NCS | Nitrogen-doped carbon | 43.6 | 7:1.5:1.5(AM : CA : PAA) | NaPF_6_/DMC:EC | 67.4 | 979 | 3852 | Not mentioned | ~790 | 0.1 | 0.01-3.0 | ^[10]^ |
|  |  |  |  |  |  |  | 2217 |  |  |  |  |  |
| SnS/FLG | Few-layer graphene | 27.0 | 8:1:1(AM : CA : CMC) | NaClO_4_/EC:PC:FEC | 76.5 | 531 | 2284 | Not mentioned | ~520 | 0.2 | 0.01-3.0 | ^[11]^ |
|  |  |  |  |  |  |  | 1137 |  |  |  |  |  |
| SnS/rGO | Reduced graphene oxide | 31.4 | 8:1:1(AM : CA : NaAlg) | NaClO_4_/EC:DEC:FEC | 68.0 | 832 | 3293 | 0.7 | ~450 | 0.2 | 0.01-3.0 | ^[12]^ |
|  |  |  |  |  |  |  | 1603 |  |  |  |  |  |
| SnS/G + C | Reduce graphene oxide/  hard carbon | 23.5 | 9.8 : 0.2(AM: CMC) | NaClO_4_/EC:DEC:FEC | 78.2 | 765 | 3271 | 2.0 | ~765 | 0.05 | 0.01-2.0 | ^[13]^ |
|  |  |  |  |  |  |  | 1539 |  |  |  |  |  |
| TNC/SnS@C | Amorphous carbon | 26.6 | 8:1:1(AM: CA : CMC) | NaClO_4_/EC:DMC:FEC | 68.7 | 698 | 3009 | 1.0 | ~500 | 0.2 | 0.01-2.5 | ^[14]^ |
|  |  |  |  |  |  |  | 1492 |  |  |  |  |  |
| SnS/C | Carbon spheres | 44.0 | 8:1:1(AM: CA : CMC) | NaClO_4_/EC:DMC:FEC | ~75.0 | 758 | 2917 | N/A | 568 | 0.05 | 0.01-2.9 | ^[15]^ |
|  |  |  |  |  |  |  | 1660 |  |  |  |  |  |
| SnS–C | Carbon | 20.0 | 7:2:1(AM : CA : PAA) | NaPF_6_/EC:DEC | 66.0 | 861 | 3905 | 2.0 | Not mentioned | 0.02 | 0.01-2.0 | ^[16]^ |
|  |  |  |  |  |  |  | 1867 |  |  |  |  |  |
| SnS/C | Macroporous carbon | 52.3 | 7:2:1(AM : CA : CMC) | NaClO_4_/EC:DEC:FEC | 76.0 | 613 | 2103 | 1.0 | ~400 | 0.1 | 0.01-3.0 | ^[17]^ |
|  |  |  |  |  |  |  | 1238 |  |  |  |  |  |
| SnS/C NFM | Carbon | 40.9 | 7:2:1(AM : CA : CMC) | NaClO_4_/EC:PC:FEC | 73.6 | 500 | 1911 | Not mentioned | ~390 | 0.2 | 0.01-2.5 | ^[18]^ |
|  |  |  |  |  |  |  | 1036 |  |  |  |  |  |
| SnS/CNTs | CNTs | 34.5 | 8:1:1(AM : CA : PVDF) | NaClO_4_/EC:DEC:FEC | ~49.0 | ~810 | 3133 | 1.0 | ~460 | 0.05 | 0.01-2.5 | **^[19]^** |
|  |  |  |  |  |  |  | 1562 |  |  |  |  |  |
| CMF/SnS-NS | carbon-coated | 58.6 | 8:1:1(AM: CA : PVDF) | NaClO_4_/EC:PC:FEC | 60.0 | 544 | 1749 | Not mentioned | ~320 | 0.05 | 0.01-3.0 | ^[20]^ |
|  |  |  |  |  |  |  | 1083 |  |  |  |  |  |
| SnS/rGO/SnS | rGO | 34.3 | 7:2:1(AM: CA : PVDF) | NaClO_4_/EC:PC:FEC | 58.8 | 908 | 3488 | Not mentioned | ~690 | 0.05 | 0.05-3.0 | ^[21]^ |
|  |  |  |  |  |  |  | 1722 |  |  |  |  |  |
| CN/SnS | Carbon coated | 31.2 | 8:1:1(AM : CA : PVDF) | NaClO_4_/EC:DMC:FEC | 53.0 | 801 | 3327 | 1.03 | Not mentioned | 0.1 | 0.01-3.0 | ^[22]^ |
|  |  |  |  |  |  |  | 1695 |  |  |  |  |  |
| SnS/A-MWCNTs | Acid-treated multiwalled CNTs | 40.0 | 7:2:1(AM : CA : PVDF) | NaPF_6_/2G | 90.0 | ~1300 | 4748 | 2.2 | ~1200 | 0.3 | 0.01-2.7 | ^[23]^ |
|  |  |  |  |  |  |  | 2438 |  |  |  |  |  |

* Conductive additives are abbreviated as CA

The density information of the material is as follows:

Active material (AM): *ρ_SnS_* = 5.22 g cc^–1^, *ρ_graphite_* = 2.27 g cc^–1^, *ρ_CNT_* = 1.3 g cc^–1^, *ρ_rGo_* = 1.82 g cc^–1^, *ρ_MXene_* = 3.7 g cc^–1^, *ρ_graphene_* = 1.82 g cc^–1^, *ρ_carbon_* = 1.82 g cc^–1^, *ρ_carbon sphere_* = 2.1 g cc^–1^

**Supplementary References**

[1] H. Laufen, S. Klick, H. Ditler, K. L. Quade, A. Mikitisin, A. Blomeke, M. Schutte, D. Wasylowski, M. Sonnet, L. Henrich, A. Schwedt, G. Stahl, F. Ringbeck, J. Mayer, D. U. Sauer, *Cell Rep.* **2024**, *5*, 101945.

[2] H. Wang, D. Huang, Z. Yan, Q. Pan, F. Zheng, Y. Huang, Q. Li, *J. Alloys Compd.* **2020**, *821*, 153551.

[3] J. Zhao, G. Wang, R. Hu, K. Zhu, K. Cheng, K. Ye, D. Cao, Z. Fan, *J. Mater. Chem. A* **2019**, *7*, 4047.

[4] M. S. Wang, H. Xu, Z. L. Yang, H. Yang, A. M. Peng, J. Zhang, J. C. Chen, Y. Huang, X. Li, G. Z. Cao, *ACS Appl. Mater. Interfaces* **2019**, *11*, 41363.

[5] C. C. J. Lee, H. Lee, S. Ma, J. W. Tan, G. Jang, S. G. Shim, Y. S. Park, J. Yun, D. W. Kim, J. Moon, *Adv. Energy Mater.* **2022**, *12*, 2103138.

[6] C. Zhu, P. Kopold, W. Li, P. A. V. Aken, J. Maier, Y. Yu, *Adv. Sci.* **2015**, *2*, 1500200.

[7] Y. Wang, J. Li, P. Song, J. Yang, Z. Gu, T. Wang, C. Wang, *J Colloid Interface Sci* **2023**, *636*, 255.

[8] S. -H. Yu, A. Jin, X. Huang, Y. Yang, R. Huang, J. D. Brock, Y. -E. Sung, H. D. Abruña, *RSC Adv.* **2018**, *8*, 23847.

[9] Y. Chen, B. Wang, T. Hou, X. Hu, X. Li, X. Sun, S. Cai, H. Ji, C. Zheng, *Chin. Chem. Lett.* **2018**, *29*, 187.

[10] S. Feng, L. Ma, J. Lin, X. Lu, L. Xu, J. Wu, X. Yan, X. Fan, *Electrochim. Acta* **2021**, *387*, 138535.

[11] D. Cheng, L. Yang, R. Hu, J. Liu, M. Zhu, *Energy Environ. Mater.* **2021**, *4*, 229.

[12] J. Li, X. Zhao, Z. Zhang, *J Colloid Interface Sci* **2017**, *498*, 153.

[13] Y. C. Lu, C. Ma, J. Alvarado, N. Dimov, Y. S. Meng, S. Okada, *J. Mater. Chem. A* **2015**, *3*, 16971.

[14] H. Ding, Y. Wu, Y. Xia, T. Yang, Z. Hu, Q. Chen, G. Yue, *J. Mater. Sci.* **2022**, *57*, 6308.

[15] J. Wang, Y. Lu, N. Zhang, X. Xiang, J. Liang, J. Chen, *RSC Adv.* **2016**, *6*, 95805.

[16] L. Wu, H. Lu, L. Xiao, J. Qian, X. Ai, H. Yang, Y. Cao, *J. Mater. Chem. A* **2014**, *2*, 16424.

[17] P. Xue, N. Wang, Y. Wang, Y. Zhang, Y. Liu, B. Tang, Z. Bai, S. Dou, *Carbon* **2018**, *134*, 222.

[18] H. Yan, M. Yang, L. Liu, J. Xia, Y. Yuan, J. F. Liu, Y. Zhang, S. Nie, X. Wang, *J. Alloys Compd.* **2020**, *843*, 155899.

[19] S. Zhang, L. Yue, H. Zhao, Z. Wang, J. Mi, *Mater. Lett.* **2017**, *209*, 212.

[20] H. -R. Yang, Y. Yang, H. Seo, K. Kim, H. S. Lee, J. Lee, J.-H. Kim, *Appl. Surf. Sci.* **2021**, *544*, 148837.

[21] G. Liu, D. Sun, X. Li, J. Liu, Y. Zhang, W. Yuan, D. Guo, N. Wu, X. Liu, *J. Mater. Sci.* **2020**, *55*, 14477.

[22] H. Yin, L. Jia, H. Li, A. Liu, G. Li, Y. Zhu, J. Huang, M. Cao, Z. Hou, *J. Energy Storage* **2023**, *65*, 107354.

[23] J. Choi, N. R. Kim, K. Lim, K. Ku, H. J. Yoon, J. G. Kang, K. Kang, P. V. Braun, H.-J. Jin, Y. S. Yun, *Small* **2017**, *13*, 1700767.
